# Comparison of k-mer-based *de novo* comparative metagenomic tools and approaches

**DOI:** 10.20517/mrr.2023.26

**Published:** 2023-07-20

**Authors:** Alise Jany Ponsero, Matthew Miller, Bonnie Louise Hurwitz

**Affiliations:** ^1^Human Microbiome Research Program, University of Helsinki, Helsinki 00290, Finland.; ^2^Department of Biosystems Engineering, The University of Arizona, Tucson, AZ 85721, USA.; ^3^BIO5 Institute, The University of Arizona, Tucson, AZ 85721, USA.

**Keywords:** *De-novo* comparative metagenomics, metagenomes, k-mers

## Abstract

**Aim:** Comparative metagenomic analysis requires measuring a pairwise similarity between metagenomes in the dataset. Reference-based methods that compute a beta-diversity distance between two metagenomes are highly dependent on the quality and completeness of the reference database, and their application on less studied microbiota can be challenging. On the other hand, *de-novo* comparative metagenomic methods only rely on the sequence composition of metagenomes to compare datasets. While each one of these approaches has its strengths and limitations, their comparison is currently limited.

**Methods:** We developed sets of simulated short-reads metagenomes to (1) compare k-mer-based and taxonomy-based distances and evaluate the impact of technical and biological variables on these metrics and (2) evaluate the effect of k-mer sketching and filtering. We used a real-world metagenomic dataset to provide an overview of the currently available tools for *de novo* metagenomic comparative analysis.

**Results:** Using simulated metagenomes of known composition and controlled error rate, we showed that k-mer-based distance metrics were well correlated to the taxonomic distance metric for quantitative Beta-diversity metrics, but the correlation was low for presence/absence distances. The community complexity in terms of taxa richness and the sequencing depth significantly affected the quality of the k-mer-based distances, while the impact of low amounts of sequence contamination and sequencing error was limited. Finally, we benchmarked currently available *de-novo* comparative metagenomic tools and compared their output on two datasets of fecal metagenomes and showed that most k-mer-based tools were able to recapitulate the data structure observed using taxonomic approaches.

**Conclusion:** This study expands our understanding of the strength and limitations of k-mer-based *de novo* comparative metagenomic approaches and aims to provide concrete guidelines for researchers interested in applying these approaches to their metagenomic datasets.

## INTRODUCTION

The advent of modern metagenomics has led to the generation of massive amounts of genomic data allowing the characterization of microbes’ diversity and their function in ecosystems. Comparative metagenomics aims to explore the similarities and differences of microbial communities by comparing metagenomes to one another. These studies generally measure the distance between each pair of metagenomes in order to investigate the impact of an ecological condition on the composition of microbial communities. By computing distances between communities, comparative metagenomic tools also provide a way to cluster similar metagenomes together or, on the contrary, distinguish distinct communities. These techniques can be used to retrieve similar metagenomes using a query metagenome or to classify a metagenome based on characteristics. For whole genome shotgun datasets, these comparisons can be achieved by measuring the similarity of the samples in terms of their taxonomic or functional diversity. These approaches require the annotation of metagenomic datasets using taxonomic or functional reference databases. On the other hand, *de novo* comparative metagenomic approaches compare metagenomic samples based on their sequence content only. Using these approaches, the similarity between datasets is measured by evaluating the proportion of shared sequences using the entire dataset, compared to Reference-based methods that can be limited by incomplete or biased reference databases (reviewed in Comin *et al*. 2021^[1]^).

Historically, *de novo* comparative metagenomic tools using WGS have relied on two distinct approaches: read-based and k-mer-based comparisons. While the first studies used alignment-based algorithms such as BLAST^[2]^ for comparing reads to one another, the ever-increasing size and number of metagenomic datasets quickly required more computationally efficient algorithms. As a result, several approaches emerged to retrieve the number of shared reads between two samples and compute a distance based on this measure. Compareads^[3]^ and its successor Commet^[4]^ approximate the read similarity between each pair of metagenomes to estimate the number of shared reads. However, these algorithms are computationally intensive and are difficult to scale to modern-size metagenomic datasets.

Instead of comparing datasets at the read level, another approach is to consider the dataset using a bag-of-word model, where a metagenomic dataset can be considered as a text composed of DNA words of length *k* (referred to as k-mers). This approach relies on three core tenets: (1) closely related organisms share k*-*mer profiles and cluster together, making taxonomic assignment unnecessary^[5]^; (2) k*-*mer frequency is correlated with the abundance of an organism^[6]^; and (3) k*-*mers of sufficient length can be used to distinguish specific organisms^[7]^. Hence, k-mers spectra can be used to differentiate between samples. The most simple and effective approach is the comparison of metagenomic datasets by calculation of pairwise distances between datasets on the basis of their composition of k*-*mers. These approaches first count the number of k-mers in the datasets using different algorithms, then calculate a dissimilarity metric between pairs of samples based on their k-mer count frequencies.

Importantly, the all-vs-all comparison of an ever-growing number of metagenomic samples, each composed of millions of reads and billions of k-mers, provides a complex challenge in terms of computation time and resources. Different approaches have been used to reduce the computing costs of such large-scale analyses. Some tools approximate the real similarity distance between metagenomes using subsampling or sketching. This approach was notably used in the k-mer-based tool MASH^[8]^ designed for an easy and fast *de novo* comparison of genomes and metagenomes. On the other hand, calculating an actual similarity distance between samples is possible and scalable when architectures such as High-Performance Computing or Hadoop clusters are used^[9,10]^.

In recent years, several bioinformatic tools that perform k-mer-based *de novo* comparative metagenomics have been released; however, it is not clear how these tools and metrics compare with each other. For biologists and domain experts to choose a tool, it is important to understand the limitations and pitfalls associated with each approach. To meet this need, we developed sets of simulated metagenomes that allowed us to (1) thoroughly assess the relationship between k-mer-based and taxonomy-based distances and evaluate the impact of technical and biological variables on these metrics, in particular, the effect of sequencing depth, sequencing technology, metagenomic contamination and community diversity; (2) evaluate the effect of sketching and filtering methods; and (3) provide an overview of the currently available tools for large-scale *de novo* metagenomic comparative analysis.

## METHODS

### k-mer-based tools for *de novo* metagenomic analysis

Each of the tools evaluated in this study was installed from the recommended source following the authors’ instructions. When tools were available from several sources, Bioconda was preferred due to simplified dependency management. Tools that could not be obtained through Bioconda were directly cloned from GitHub or Sourceforge.

All tools were run in a SLURM High Performance Computing (HPC) environment. The standard running conditions were four cores and 24 GB of memory. If a tool had higher memory requirements, it received more memory but was limited to four threads to keep runtime comparisons consistent. If a tool supported paired-end reads, the R1 and R2 files were used; otherwise, only the R1 files were used. If a tool allowed for cluster computing commands, they were used, with subjobs limited to four cores and 24 GB of memory. Pre-processing, such as read filtering and trimming, was not included in the runtimes. A k-mer size of *k* = 31bp was used for all tools except CAFE which used a k-mer size of 5bp, and all tools were run with the default options, excluding threads, memory, cluster computing, and k-mer size options.

### Simulated datasets

This study leverages four distinct simulated datasets: (1) SimSet 1 to assess technical effects; (2) SimSet 2 to mimic low abundance contamination effects; (3) SimSet 3 to assess the impact of microbial community richness; and (4) SimSet 4 to assess the impact of taxonomic diversity.

All simulated datasets were generated using InSilicoSeq v1.5.4^[11]^. Briefly, this tool uses an error model of per-base quality (Phred) scores using Kernel Density Estimation, trained on real sequencing reads, and is able to generate reads with realistic quality score distributions for several sequencing platforms, including MiSeq, HiSeq, and NovaSeq^[11,12]^. All simulated metagenomes were generated from complete bacterial and archaeal genomes downloaded from RefSeq in November 2022^[13]^.

#### SimSet 1: technical effects

The simulated dataset 1 (SimSet 1) addressed technical variations between metagenomic datasets and, more specifically, differences in sequencing technology and sequencing depth. The dataset is composed of 100 simulated metagenomes, each containing 25 bacteria species picked randomly from a list of 40 possible organisms that were randomly selected from complete genomes available in the RefSeq database^[13]^. The relative abundance of each of the 25 organisms in each simulated metagenome was obtained from a log-normal distribution.

From the generated relative abundance profiles, InSilicoSeq was used to simulate metagenomes of increasing sequencing depth (50K, 100K, 500K, 1M, 5M, 10M, and 50M paired reads), using MiSeq, HiSeq, and NovaSeq error profiles.

#### SimSet 2: human/PhiX contamination effect

The simulated dataset 2 (SimSet 2) aims to evaluate the impact of low and high human DNA and low Phi X174 phage contamination in metagenomes. The low human contamination experiment leverages the relative abundance profiles used for the SimSet 1, with the random addition of 0 to 2% of human reads. On the other hand, the high contamination experiment uses the SimSet 1 relative abundance profile but with the random addition of 10% to 25% human reads. The PhiX contamination experiment uses the same relative abundance profiles, with a random addition of 0 to 2% Phi X174 reads.

From these contaminated relative abundance profiles, InSilicoSeq was used to simulate metagenomes of increasing sequencing depth (50K, 100K, 500K, 1M, 5M, 10M, and 50M paired reads), using HiSeq error profiles.

#### SimSet 3: community richness effect

The simulated dataset 3 (SimSet 3) aims to evaluate the impact of increasing species richness. The dataset is composed of 5 sets of 100 simulated metagenomes each, containing 5, 25, 50, 100, or 500 bacterial species picked randomly from a list of 10, 40, 80, 130 or 530 possible organisms, respectively. The relative abundance of each organism in each simulated metagenome was obtained from a log-normal distribution.

From the generated relative abundance profiles, InSilicoSeq was used to simulate metagenomes of increasing sequencing depth (50K, 100K, 500K, 1M, 5M, 10M, and 50M paired reads), using HiSeq error profiles.

#### SimSet 4: community taxonomic richness effect

The simulated dataset 4 (SimSet 4) aims to evaluate the impact of increasing taxonomic diversity. The dataset is composed of 3 sets of 100 simulated metagenomes each, containing 50 bacterial species picked randomly from a list of 80 possible organisms belonging to the same taxonomic class (Actinomyces) or from the same taxonomic family (Mycobacteriaceae). An additional dataset was generated similarly but including all possible taxonomic classes. The relative abundance of each organism in each simulated metagenome was obtained from a log-normal distribution.

From the generated relative abundance profiles, InSilicoSeq was used to simulate metagenomes of increasing sequencing depth (50K, 100K, 500K, 1M, 5M, 10M, and 50M paired reads), using HiSeq error profiles.

### Beta-diversity distances between simulated metagenomes

Three distinct types of distance metrics were computed on the simulated metagenomes:

The expected taxonomic beta-diversity distances (Bray-Curtis and presence/absence Jaccard distances) were computed on the simulated samples’ taxonomic abundance profiles using the Vegan R package^[14]^.

Read-based taxonomic profiles were obtained using Kraken2^[15]^ and Bracken^[16]^ on the simulated metagenomes using the “Standard plus protozoa & fungi database” (from https://benlangmead.github.io/aws-indexes/k2 on 05.2021). The read-based taxonomic beta-diversity distances (Bray-Curtis and presence/absence Jaccard distances) were computed on the simulated samples’ taxonomic abundance profiles using the Vegan R package.

k-mer-based beta-diversity distances were computed using Simka (Bray-Curtis and presence/absence Jaccard distances), with controlled k-mer length. The minimum abundance k-mer filter was set to 2 and the maximum abundance k-mer filter to 999999999^[10]^.

Spearman correlations between the different types of beta-diversity distances were assessed using the Stats R package v3.6.2.

### Effect of sketched k-mer distances

A simulated dataset of 100 simulated metagenomes composed of 25 organisms each was generated using InSilicoSeq for a sequencing depth of 5 million reads and with the HiSeq error model. The exact k-mer-based Bray-Curtis and presence/absence Jaccard distances were obtained for determined k-mer lengths using Simka with the default filtering parameter. Sketched k-mer profiles and distances were obtained using SimkaMin^[17]^ at determined k-mer and sketch sizes.

The absolute difference between the exact and sketched k-mer distance was calculated for each sample pair comparison. The correlation between the expected Bray-Curtis distances on the simulated taxonomic profiles and the sketched k-mer-based distances was calculated using a Spearman correlation.

### Minimum and maximum abundance k-mer filter effects

For this experiment, a simulated dataset of 100 simulated metagenomes composed of 25 organisms each was generated using InSilicoSeq for a sequencing depth of 5 million reads and using an HiSeq error model. K-mer-based Bray-Curtis and presence/absence Jaccard distances were obtained for a determined k-mer length using Simka without a k-mer filter. Distances also were computed on the same simulated metagenome dataset using the minimum k-mer abundance or maximum k-mer abundance parameter from Simka. The absolute difference between the unfiltered and filtered k-mer distance was calculated for each sample pair comparison. The correlation between the expected Bray-Curtis distances on the simulated taxonomic profiles and the filtered k-mer-based distances was calculated using a Spearman correlation.

### Benchmark on infant and mother metagenomic dataset

Publicly available fecal metagenomes from infants and pregnant mothers were retrieved from the European Nucleotide Archive (ENA Bioproject ID: PRJEB52774). The sample collection and sequencing are described in a previously published study^[18]^. Sequences were trimmed and quality filtered using FastQC v0.11.9 and Trim Galore v0.6.6 with default parameters. Quality-filtered sequences were screened to remove human read sequences using Bowtie2 v2.4.2 against the Human genome (Human Build 38, patch release 7). After quality control and human read filtering, infant fecal metagenomes containing less than 10 million paired-end reads and mother fecal metagenomes with less than 20 million paired-end reads were discarded. Taxonomic profiling of the metagenomic samples was performed using Kraken2 v2.1.1^[15]^ against the HumGut database^[19]^, and Bracken v2.6.1 was run on Kraken2 outputs^[16]^. PCoA visualization of the distances computed between sample pairs was generated using the ecodist R package v2.0.9.

Before hierarchical clustering of the samples, low-abundance species (< 0.01% relative abundance and < 0.1% prevalence) were filtered out. Then, the dataset was transformed into relative abundances, and a distance matrix was calculated from the transformed data using the Bray-Curtis or presence/absence using the Ecodist function. Hierarchical clustering was done with the function *hclust* and with the Wald.D2 method. Clusters’ purity was calculated as follows: (1) each cluster was assigned to the sample group, which is most frequent in the cluster; (2) the accuracy of this assignment was measured by counting the number of correctly assigned samples; and (3) dividing the accuracy by the total number of samples.

PERMANOVA testing was performed using the adonis2 function from the vegan R package using 999 permutations.

## RESULTS

### Comparing k-mer-based and taxonomy-based analysis

**Figure 1:**
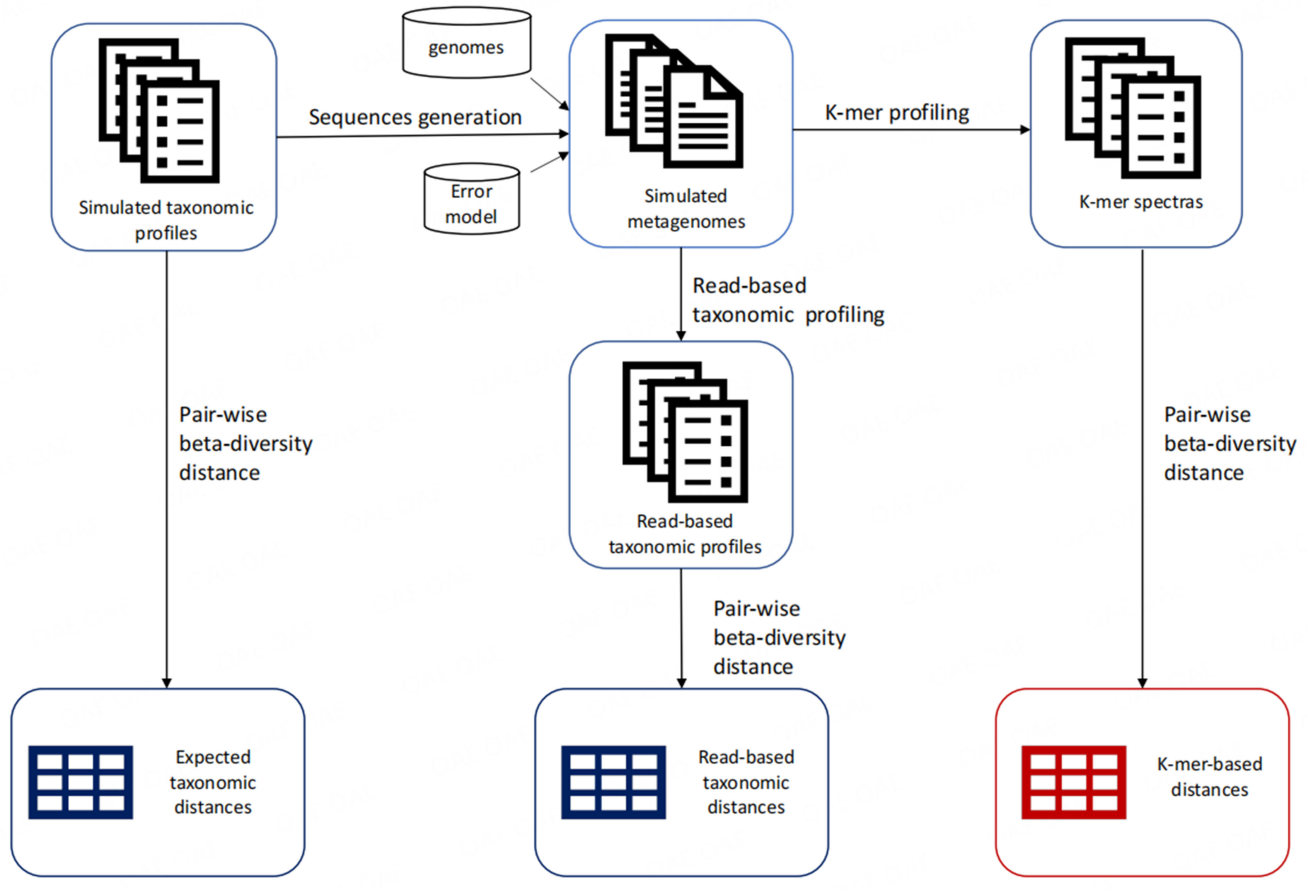
Overview of simulated experiments. Simulated metagenomic reads were generated using InSilicoSeq. The k-mer spectra were obtained using Simka and read-based profiles using Kraken2 and Bracken.

To assess and compare beta-diversity distances obtained using Reference-based and k-mer-based approaches, four simulated short-reads metagenomic datasets were generated. Each dataset was composed of 100 metagenomes, and each sample had a known taxonomic composition and relative abundance profile. Pairwise beta-diversity metrics were computed between all pairs of samples in the dataset using the true taxonomic profile at the species level and is referred to as the “expected taxonomic-based” beta-diversity metric. Using the generated sample taxonomic composition and profiles, simulated metagenomic reads were generated with a given sequencing depth and sequencing error model. The k-mer-based beta-diversity distances between each pair of simulated metagenomes were assessed using Simka^[10]^ and are referred to as “k-mer-based” beta-diversity metrics. Finally, the simulated metagenomes were profiled using the read classifier Kraken2 and Bracken. The read counts obtained were used to compute a “read-based taxonomic” beta-diversity metric at the species level. It is important to note that because all genomes used to generate the mock communities are present in the Kraken2 database, the impact of unknown taxa in metagenomes is not investigated in this experiment. The correlation between the beta-diversity metrics for the same sample pairs was measured using a Spearman correlation. Figure 1 provides an overview of the simulated experiment.

### Technical effects

We first evaluated the correlation between taxonomic-based beta-diversity and k-mer-based metrics in simple simulated metagenomes and assessed the potential impact of technical variables such as sequencing technology and sequencing depth. A simulated dataset (SimSet 1) of 100 simulated metagenomes composed of 25 bacterial species was generated for three different sequencing technologies (HiSeq, MiSeq, and NovaSeq) and at different sequencing depths (50K, 100K, 500K, 1M, 5M, 10M, and 50M paired reads). The “expected taxonomic-based” beta-diversity distances (Bray-Curtis and presence/absence Jaccard distance) were computed at the species level between each pair of samples using the true taxonomic profiles used to generate the simulated metagenomes. The same beta-diversity distances were computed on the simulated metagenomes’ k-mer composition using Simka at different k-mer lengths (10, 15, 20, 25, and 30)^[10]^. The correlation between expected taxonomic and k-mer-based beta-diversity distances was assessed for each setting using Spearman correlations.

**Figure 2:**
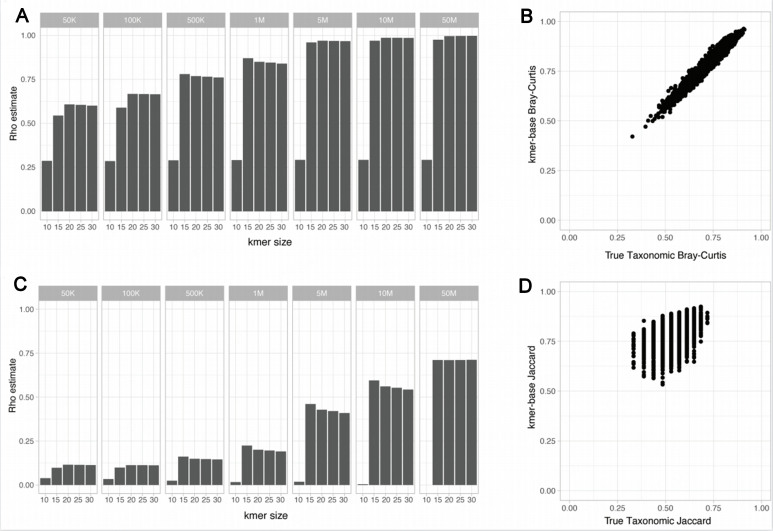
Impact of Sequencing technology, sequencing depth and k-mer length on the correlation between expected taxonomic and k-mer-based beta-diversity metric. (A) Spearman correlations between the expected taxonomic and k-mer-based Bray-Curtis distance using the HiSeq sequencing error model; (B) Expected taxonomic against the k-mer-based Bray-Curtis distances (*k* = 30bp) obtained for a simulated dataset of 100 metagenomes simulated at a sequencing depth of 5 million paired reads using the HiSeq sequencing error model; (C) Spearman correlations between the expected taxonomic and k-mer-based presence/absence Jaccard distance using the HiSeq sequencing error model; (D) Expected taxonomic against the k-mer-based presence/absence Jaccard distances (*k* = 30bp) obtained for a simulated dataset of 100 metagenomes simulated at a sequencing depth of 5 million paired-reads using the HiSeq sequencing error model.

On simple communities of only 25 organisms, the expected taxonomic and k-mer-based Bray-Curtis distances are overall well correlated (rho estimate > 0.75 in most tested conditions) [Figure 2A]. The correlation is linear [Figure 2B], and both Spearman and Pearson correlations give consistent results (not shown). The correlation between expected taxonomic and k-mer-based Bray-Curtis distances is affected by both the k-mer size and sequencing depth, with the strongest correlations observed for a k-mer size above 20bp and a sequencing depth above 1 million reads [Figure 2A]. On the other hand, the sequencing technology had only a minimal impact on the observed correlations [Supplementary Figure 1].

The correlations between expected taxonomic and k-mer-based presence/absence Jaccard distances were globally poor, with a rho estimate below 0.5 in most tested conditions [Figure 2C and D]. Similar to the results for the Bray-Curtis distances, longer k-mer sizes (> 15bp) and higher sequencing depth (> 1M reads) improved the correlations with the expected Jaccard distances, while the choice of sequencing technologies only had a minimal impact [Supplementary Figure 1].

Notably, in all tested conditions, the correlations between expected taxonomic and k-mer-based distances were poor when considering shallow sequencing depth below 1M reads. Given the simple composition of the mock communities, composed of only 25 organisms each, read-based classifiers such as Kraken2 allows for a complete description of the total community richness even at the shallowest sequencing depth (50k reads). However, k-mer-based beta-diversity distances computed on shallow datasets are overestimated, with most samples-to-samples k-mer-based distances close or equal to 1 [Supplementary Figure 2].

**Figure 3:**
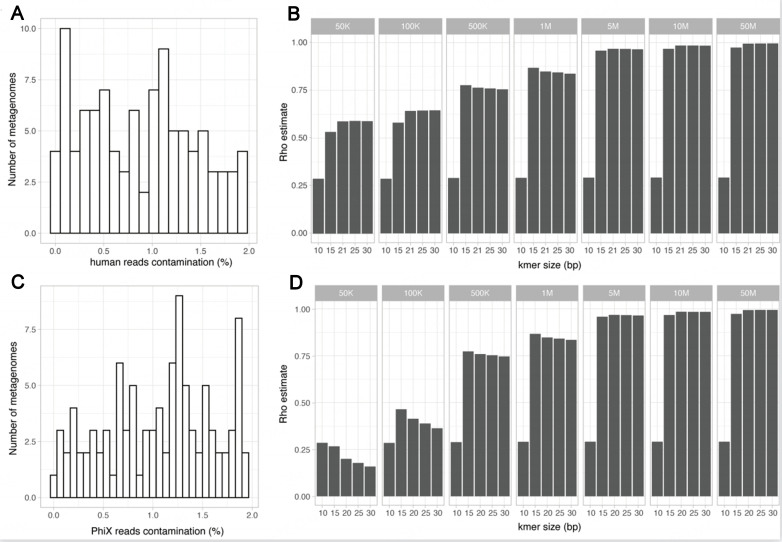
Impact of low abundance human and PhiX174 sequence contaminations on the correlation between expected taxonomic and k-mer-based beta-diversity metric. (A) Distribution of the percentage of human reads content in the simulated dataset; (B) Spearman correlations between the expected taxonomic and k-mer-based Bray-Curtis distance for the human contaminated dataset; (C) Distribution of the percentage of PhiX174 reads content in the simulated dataset; (D) Spearman correlations between the expected taxonomic and k-mer-based Bray-Curtis distance for the PhiX174 contaminated dataset.

We next assessed the impact of human DNA contamination on the observed correlations between true taxonomic and k-mer-based beta-diversity distances. A new simulated dataset (SimSet 2) composed of 100 simulated metagenomes, each containing 25 bacterial species, was created using the HiSeq error model and the same range of sequencing depth as the SimSet 1. Human reads were added randomly to the simulated metagenomes to reach a relative abundance between 0% and 2% of the total reads [Figure 3A]. The Bray-Curtis beta-diversity distances between samples were computed as previously, and the correlation between k-mer-based and expected taxonomy distances was assessed. Interestingly, the overall impact of random and low human DNA contamination on the correlations was minimal in all settings tested [Figure 3B], and similar results were obtained for the presence/absence Jaccard index (not shown).

Using similar settings, we created a simulated dataset mimicking low contamination by the *E.coli* phage Phi X174, classically used to spike metagenomic sequencing runs. In this dataset, the Phi X174 reads accounted for less than 2% of the total reads [Figure 3C]. As for the low human read contamination, this low Phi X174 read contamination had a minimal impact on the correlations between k-mer-based and expected taxonomy distances [Figure 3D].

While low contaminations from both human and Phi X174 sequences had limited impact on the k-mer-based beta-diversity distance estimations, a noticeable impact could be seen in the case of high contamination settings. Using a simulated dataset mimicking high contamination of human DNA (10% to 25% of the reads), we observed a degraded correlation between the k-mer-based and true taxonomy-based Bray-Curtis [Supplementary Figure 3].

### Community composition effects

While the impact of technical effects was assessed on a simple community composed of 25 bacterial species, most real-world metagenomes are characterized by higher species richness. We next assessed how k-mer-based beta-diversity distances compare to expected beta-diversity distances on more complex artificial communities. The SimSet 3 is composed of five datasets of 100 artificial metagenomes each, composed of an increasing number of bacterial taxa (5, 25, 50, 100, and 500 organisms), and simulated reads were generated to simulate a range of sequencing depth (500K, 1M, 5M, and 10M paired reads) using a HiSeq error model.

**Figure 4:**
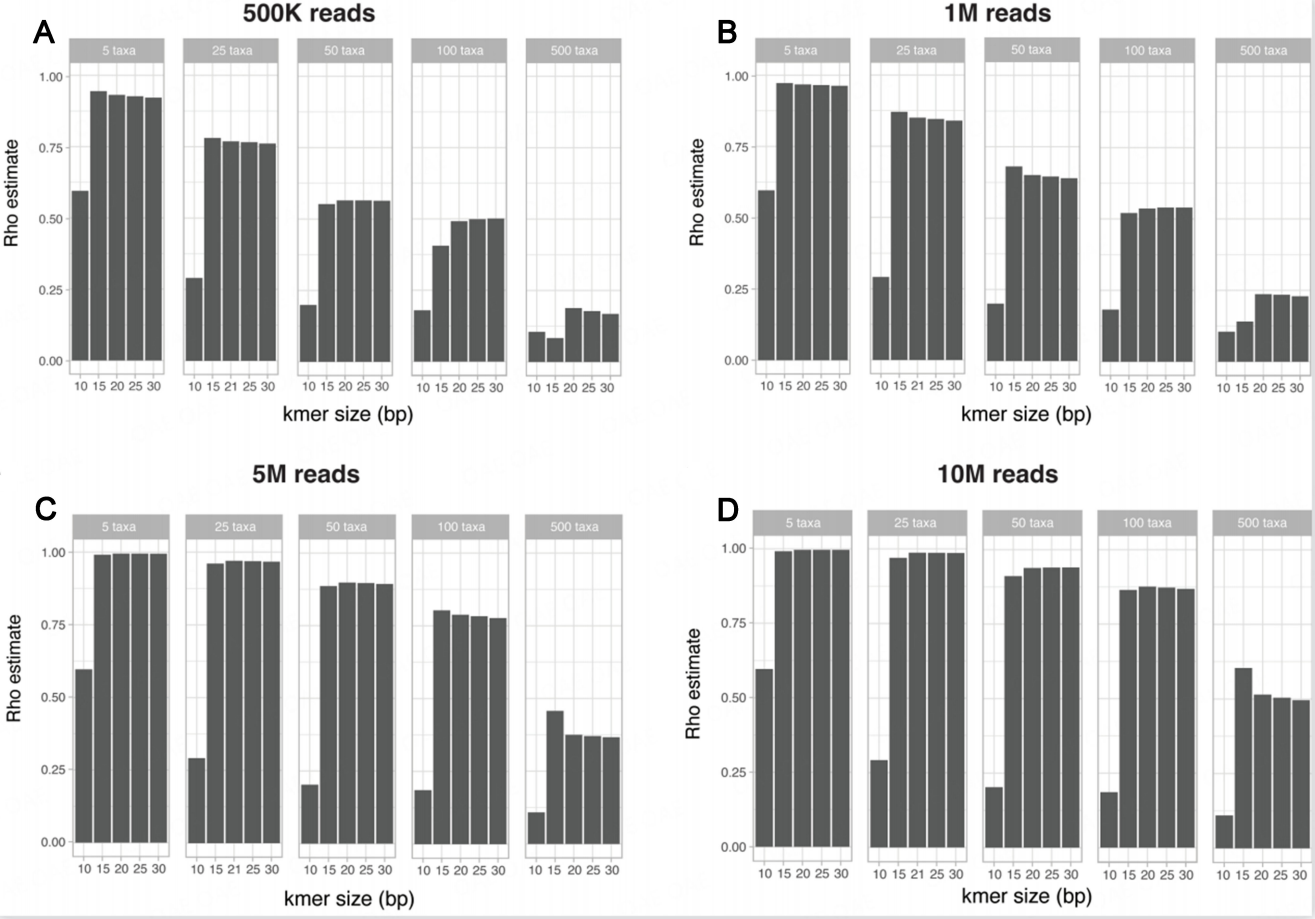
Impact of increasing community species richness on the correlation between expected taxonomic and k-mer-based beta-diversity metric. Spearman correlations between the expected taxonomic and k-mer-based Bray-Curtis distance for simulated communities containing an increasing number of taxa, for a simulated sequencing depth of (A) 500K paired-reads; (B) 1 Million paired-reads; (C) 5 Million paired-reads; or (D) 10 Million paired-reads.

When considering communities with increasing richness, the observed correlations between the expected taxonomic and k-mer-based Bray-Curtis metrics are more susceptible to shallow sequencing depth effects. While expected taxonomic and k-mer-based Bray-Curtis metrics between simple communities are well correlated with each other at shallow sequencing depth [Figure 4A and B], the correlation is decreased when considering more complex communities at the same sequencing depth [Figure 4C and D]. Strikingly, for the simulated community composed of 500 organisms, the correlation between the expected taxonomic- and k-mer-based Bray-Curtis metrics was weak (rho estimate of 0.52 of *k* = 20bp), even for a sequencing depth of 10 million reads.

A similar impact of the increasing community diversity and sequencing depth is observed for presence/absence Jaccard index, but as observed for the SimSet 1, the expected presence/absence Jaccard index and k-mer-based presence/absence Jaccard indices were globally poorly correlated in all tested situations [Supplementary Figure 4].

### Community taxonomic diversity

In the previous simulated dataset (SimSet 3), the richness of a simulated community was considered in terms of the number of different species. Next, we assessed the effect of a reduced taxonomic diversity by creating three simulated sets of 50 organisms from any bacterial class (referred to as “All taxa dataset”), only from the Actinomycetes class (referred to as “same class” dataset) or from the same Mycobacterium family (referred to as “same family” dataset). The Mycobacterium family was chosen as it contains more than 100 species, including several major human pathogens as well as numerous other environmental species. As previously, the simulated metagenomes were generated for a range of sequencing depths (500K, 1M, 5M, and 10M paired reads) using a HiSeq error model.

**Figure 5:**
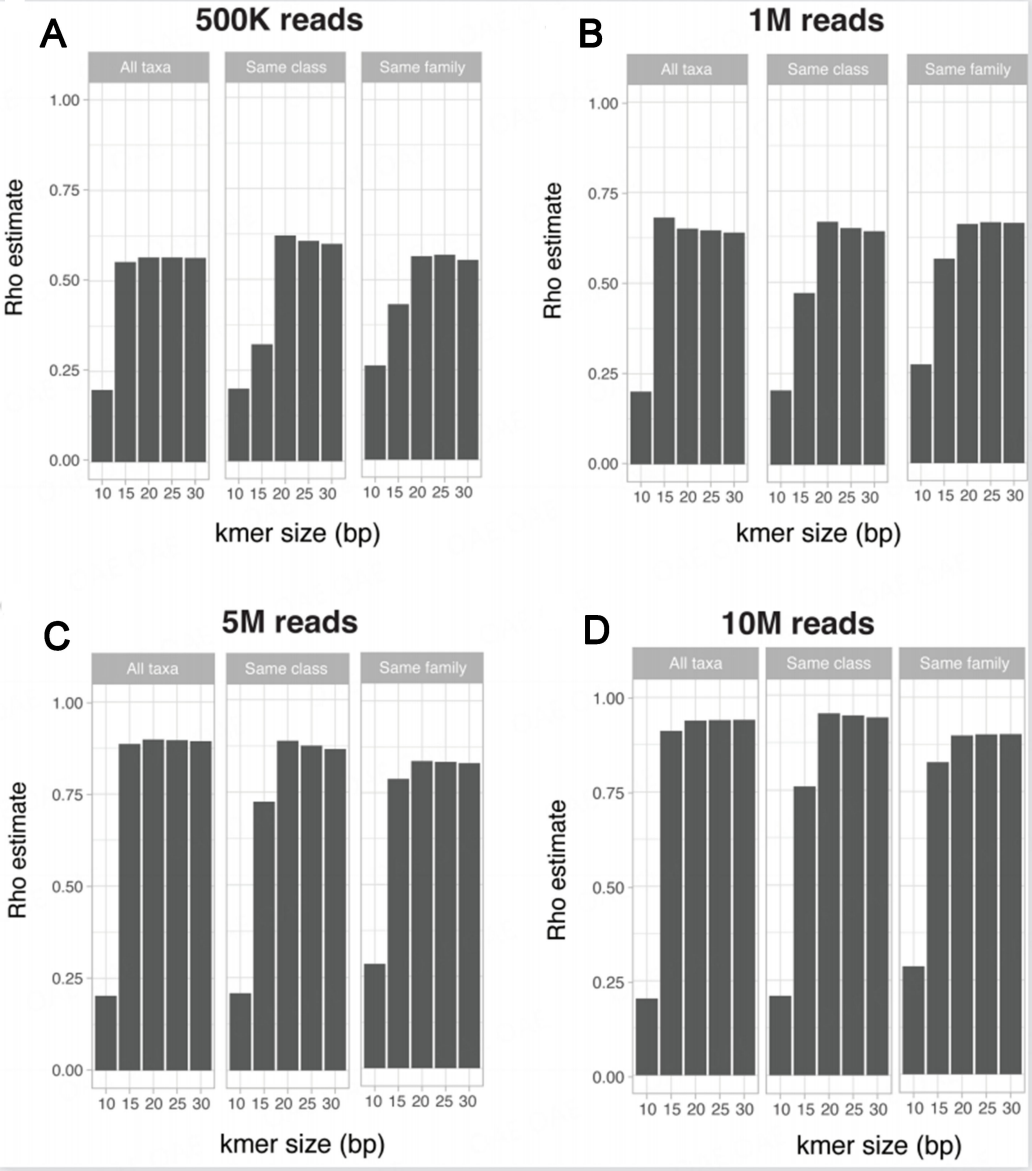
Impact of decreasing taxonomic diversity on the correlation between expected taxonomic and k-mer-based beta-diversity metric. Spearman correlations between the expected taxonomic and k-mer-based Bray-Curtis distance for simulated communities containing 50 taxa from all possible taxonomic classes (“All taxa”), from the Actinomycetes class (“Same class”) or from the Mycobacterium family (“Same family”). Simulated metagenomes were generated to simulate a sequencing depth of (A) 500K paired reads; (B) 1 Million paired reads; (C) 5 Million paired reads; or (D) 10 Million paired reads.

The correlation between the expected taxonomic Bray-Curtis and the k-mer-based Bray-Curtis metrics were comparable for the three datasets at all sequencing depths. The correlation was markedly lower for a k-mer size of 15bp for the “same class” and “same family” datasets than for the “all taxa” dataset [Figure 5]. The results for the presence/absence Jaccard index were comparable between the three datasets at all tested k-mer sizes and sequencing depths [Supplementary Figure 5].

### Assessing the effect of sketching

In order to alleviate the high computational requirements necessary to compute exact k-mer counts for large-scale metagenomic datasets, dimensionality reduction approaches were proposed to obtain a simpler feature vector description of a metagenomic sample. Tools such as MASH^[8]^, SimkaMin^[17]^, HULK^[20]^, SourMash^[21]^, and kWip^[22]^ use a local sensitive hashing to randomly subsample the k-mer space of each sample, reducing the set of sequences into sketches. Beta-diversity distances such as Bray-Curtis and Jaccard indices can be estimated on such sketches, considerably reducing the time required for distance computation between samples.

**Figure 6:**
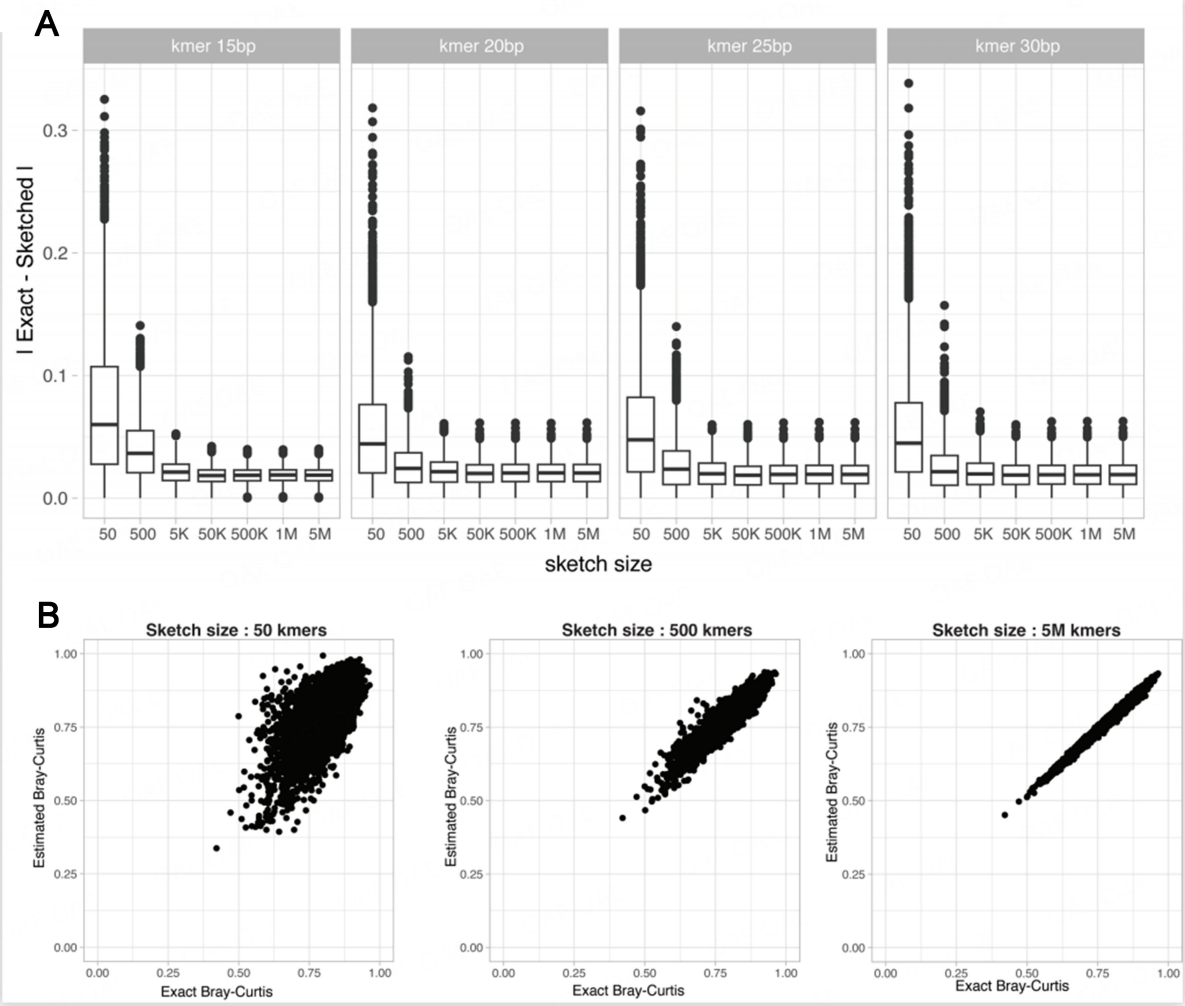
Impact of sketching k-mer on the estimation of k-mer-based Bray-Curtis distances. (A) Absolute differences between the exact k-mer-based Bray-Curtis distances and sketched Bray-Curtis distances for an increasing Sketch size; (B) Exact k-mer-based against the sketched Bray-Curtis distances (*k* = 30bp) obtained for a simulated dataset of 100 metagenomes simulated at a sequencing depth of 5 million paired reads using the HiSeq sequencing error model.

In order to assess the effect of sketching size on the precision of the k-mer-based distance, we computed the absolute difference between exact and sketched k-mer-based Bray-Curtis and presence/absence Jaccard distances obtained on a dataset of simulated metagenomes composed of 25 organisms sequenced at 5 million reads using a HiSeq error model (SimSet 1). As expected, the difference between exact k-mer and sketched Bray-Curtis indices decreased as the sketch size increased [Figure 6]. Strikingly, for all considered k-mer lengths, even small sketch sizes allowed for a reliable estimation of the indices. However, increasing the sketch size above 50K k-mers only marginally improved the estimated distance. As expected from a presence/absence distance metric, the estimation of presence/absence Jaccard distances was noisier than for the Bray-Curtis distances, even considering the large sketch size [Supplementary Figure 6].

### Assessing the impact of k-mer filtering

In order to improve upon the k-mer-based beta-diversity measures, several tools enable users to filter out or weigh k-mers for consideration in the comparison between samples. In particular, the ability to filter extremely low abundance k-mers or extremely highly abundant k-mers was proposed as a method to remove potentially erroneous k-mers due to sequencing error or to filter out contaminants in the metagenomes^[10]^. While the rationale behind the use and chosen thresholds for these filters has been mostly empirical, some tools, such as Simka, implement a default filtering of low (*n* < 2) and high abundance k-mers. In order to assess the impact of k-mer filtration on the k-mer-based distance metrics, the Bray-Curtis and presence/absence Jaccard index was computed between pairs of samples in a simulated community composed of 25 random organisms, sequenced at 5 million reads using a HiSeq error model (SimSet 1). Distances obtained on the same sample pairs before and after filtering of k-mers were compared to the expected taxonomic beta-diversity metric.

**Figure 7:**
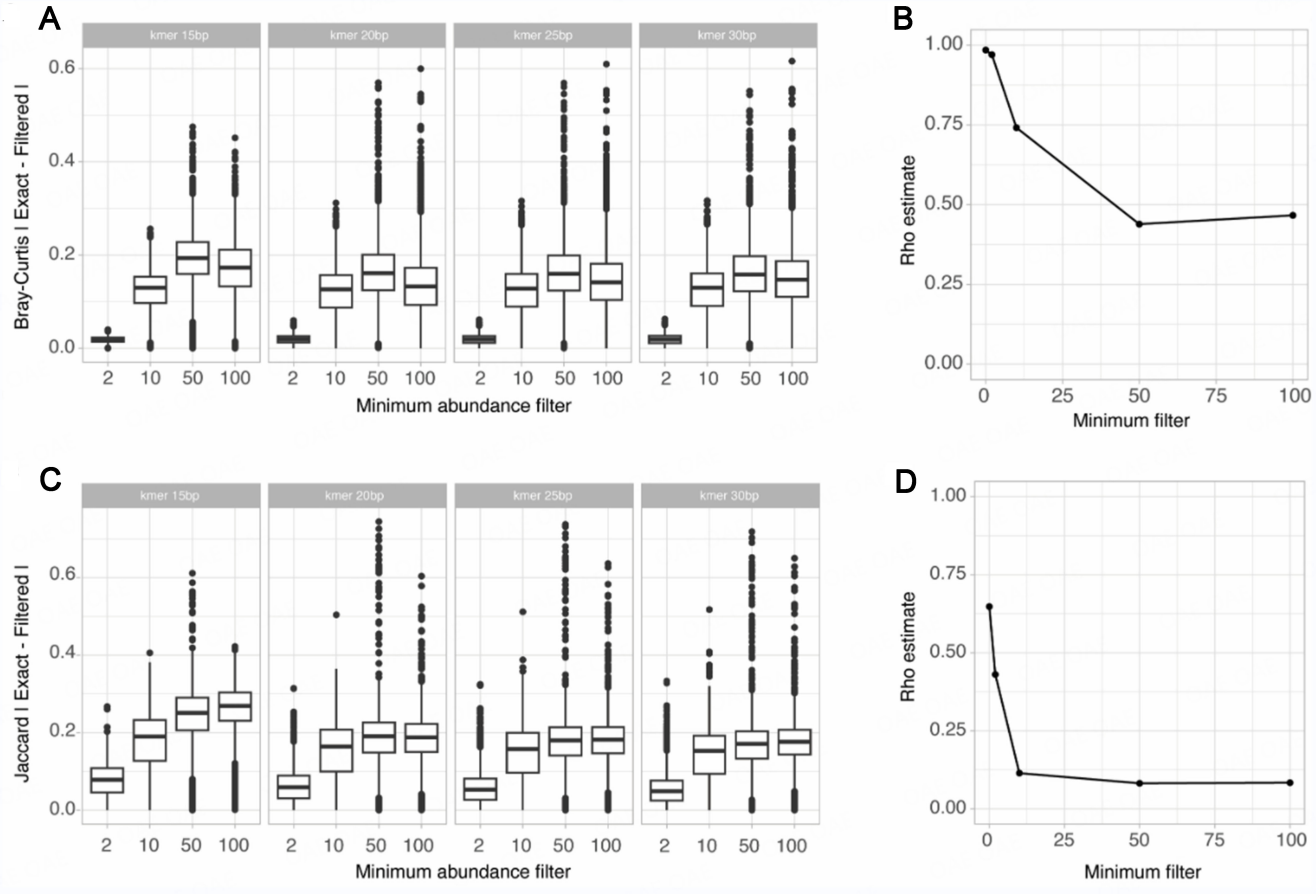
Impact of low abundance k-mer filter on the estimation of k-mer-based Bray-Curtis distances. (A) Absolute differences between the exact k-mer Bray-Curtis distances and distances after low abundance k-mer filter; (B) Spearman correlation between the expected taxonomic Bray-Curtis index and the k-mer-based Bray-Curtis index when increasing the low abundance k-mer filter; (C) Absolute differences between the exact k-mer presence/absence Jaccard distances and distances after low abundance k-mer filter; (D) Spearman correlation between the expected taxonomic presence/absence Jaccard index and the k-mer-based Jaccard index when increasing the low abundance k-mer filter.

We first assessed the impact of filtering low abundance k-mers on the Beta-diversity distances, by comparing the k-mer-based distances without filter to the same distance obtained when increasing the minimum abundance k-mer filtering threshold. As expected, the filtering of low-abundance k-mers had a more important effect on the presence/absence Jaccard indices compared to the Bray-Curtis indices [Figure 7A and B]. Importantly, for both metrics, using the minimum abundance k-mer filter degraded the correlation between the expected taxonomic and k-mer-based taxonomic distances for both metrics [Figure 7C and D].

Similarly, we assessed the impact of filtering high abundance k-mers, by comparing the k-mer-based distances without filter to the same distance obtained when increasing the maximum abundance k-mer filtering threshold. As previously, the filtering of high abundance k-mers had a more important effect on the presence/absence Jaccard indices [Supplementary Figure 7]. Expectedly, the maximum abundance filter effect depends on the k-mer size, because a larger proportion of shorter k-mer size will be filtered out for the same maximum abundance threshold.

### Benchmark of published k-mer *de-novo* comparative tools

Finally, we reviewed and compared published *de-novo* comparative metagenomic tools to assess each tool’s characteristics and usability on a real-world dataset. A total of 12 previously published *de-novo* comparative metagenomic tools were found in the literature [Table 1] between 2016 and 2020. Published tools could be grouped into three broad approaches, (1) read-based k-mer comparison tools that compare metagenomes on their read content; (2) complete k-mer spectra comparison tools that compare metagenomes on their complete k-mer content; and (3) sketched k-mer spectra tools that leverage a sketching approach to approximate k-mer-based distances. We installed and benchmarked each tool’s computing requirements in terms of CPU time and memory usage on a dataset of 30 metagenomes (74 GB). LIBRA was excluded as the tool requires a Hadoop cluster to be run^[9]^, Comparead^[3]^ was excluded since the tool was deprecated when Commet was published^[4]^, and TriageTool^[23]^ could not be installed and run as the tool was not updated for current systems. As expected, Commet, which used a read-based comparison, required a significantly longer computational time than the other k-mer-based tools. Tools relying on a sketching approach to accelerate their computation finished the pairwise comparison quickly, between 20 min to 4 h. Strikingly, Simka, which computes complete k-mer spectra finished the comparison in a comparable time with the same resources. Finally, Metafast^[24]^ required a larger memory allocation to perform this comparison and kWIP^[22]^ memory requirements were dependent on the size of the k-mer countgraph generated by khmer^[25]^. A max sketch size of 1e09 utilized 17 GB of memory, while a max sketch size of 1e10 utilized 150.58 GB of memory. Cafe was originally designed to perform k-mer-based comparisons using a small k-mer size, and the authors previously demonstrated the tool’s performance on metagenomic datasets using a k-mer size of 5bp^[26]^. In order to fairly assess the computational requirements of all tools on a similar task, we attempted to run the tool using a k-mer size of 31bp. However, these parameters required a large memory size that could not be accommodated. Presented below are therefore the computational requirements of Cafe using a k-mer size of 5pb.

**Table 1:** Summary of tools, overview of the algorithm, reference to the publication and computational resources for the pairwise comparison of 30 metagenomes

**Name**	**Algorithm summary**	**Output distance**	**Publication**	**Resources**
MASH	Sketched k-mer spectra	Presence/absence Jaccard	Ondov *et al*. 2016^[8]^	CPU: 4 Mem: 4.21 MB Runtime: 01:04:36
kWIP	Sketched k-mer spectra	Other	Murray *et al*. 2017^[22]^	CPU: 4 Mem: 150.58 GB Runtime: 03:24:55
SourMash	Sketched k-mer spectra	Other	Pierce *et al*. 2019^[21]^	CPU: 4 Mem: 88.63 MB Runtime: 02:26:23
HULK	Sketched k-mer spectra	Presence/absence Jaccard Bray-Curtis & Others	Rowe *et al*. 2019^[20]^	CPU: 4 Mem: 2.2 GB Runtime: 04:10:51
Simka-min	Sketched k-mer spectra	Presence/absence Jaccard Bray-Curtis	Benoit *et al*. 2020^[17]^	CPU: 4 Mem: 24 GB Runtime: 00:20:08
Simka	Complete k-mer spectra	Presence/absence Jaccard Bray-Curtis & Others	Benoit *et al*. 2016^[10]^	CPU: 4 Mem: 4.10 GB Runtime*: 02:37:27
Metafast	k-mer based, de Bruijn graphs	Bray-Curtis	Ulyantsev *et al*. 2016^[24]^	CPU: 4 Mem: 66.98 GB Runtime: 03:26:11
LIBRA	Complete k-mer spectra	Bray-Curtis & Others	Choi *et al*. 2019^[9]^	Not computed (requires Hadoop cluster)
Triagetool	Read-based k-mer comparison	Other	Fimerelli *et al*. 2013^[23]^	Not computed (not updated for recent systems)
Compareads	Read-based k-mer comparison	Other	Maillet *et al*. 2012^[3]^	Not computed (tool deprecated)
Commet	Read-based k-mer comparison	Other	Maillet *et al*. 2014^[4]^	CPU: 4 Mem: 1.07 GB Runtime: 17:07:14
Cafe**	Sketched k-mer spectra	Other	Lu *et al*. 2017^[26]^	CPU: 4 Mem: 6.39 GB Runtime: 00:03:53

*For tools with cluster commands, the subjobs also received four cores and 24 GB of memory. Runtime was calculated by summing the runtime of the main job and all subjobs. Each counting subjob for Simka averaged 4 GB of memory utilized; **Cafe was run using a k-mer size of 5bp.

**Figure 8:**
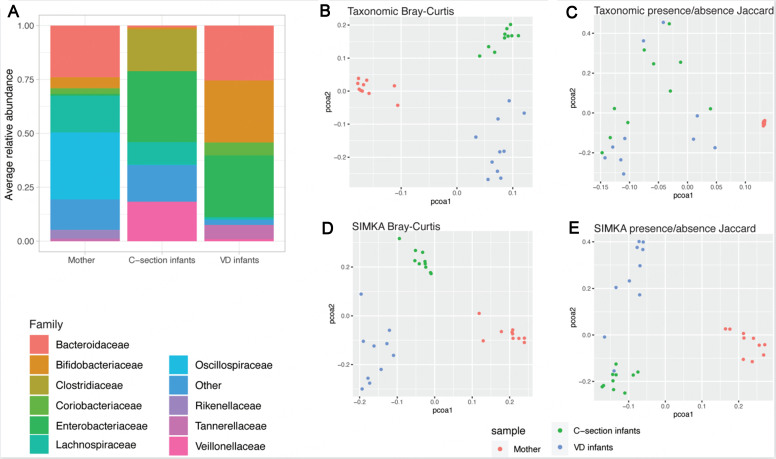
Comparison of taxonomic and k-mer-based approaches on a small dataset of infant and maternal fecal metagenomes. (A) Average composition of the samples grouped by sample origin at the Family level, taxonomic families with a prevalence below 10% and a relative abundance below 5% were grouped as “Other”; (B) PcoA of the samples on the taxonomic profiles at the species level using a Bray-Curtis distance; (C) PcoA of the samples on the taxonomic profiles at the species level using a presence/absence Jaccard distance; (D) PcoA of the samples on the k-mer spectra using a Bray-Curtis distance (E) PcoA of the samples on k-mer spectra profiles using a presence/absence Jaccard distance. VD infants: Vaginally delivered infants.

We next compared the k-mer-based tools on a clustering task using a real metagenomic dataset of 30 metagenomes from 3-week-old infant and adult fecal samples. The samples’ taxonomic profiles were obtained using a read classifier, and the dataset was visualized using a PcoA on Bray-Curtis or presence/absence Jaccard. At the taxonomic level, the dataset was composed of three distinct sample clusters, mother samples, infants born by C-Section, and infants born vaginally [Figure 8A]. Hierarchical clustering was performed on the computed distances using a ward linkage method, and the purity of the obtained clusters was calculated. The taxonomic Bray-Curtis distance allowed for a clear separation between the three types of samples (cluster purity = 1), while the presence/absence Jaccard distance separated only infants from mother samples but did not allow for a clear separation of the samples according to delivery mode (cluster purity = 0.67) [Figure 8B and C]. K-mer-based distances were computed for these samples using Simka, SimkaMin, Mash, HULK, Metafast, kWIP, and SourMash using the same k-mer size (*k* = 31bp). With complete k-mer spectra, using Simka, the data structure observed was well conserved, and samples were clearly separated as expected (cluster purity = 0.97 for Bray-Curtis, cluster purity = 0.9 for presence/absence Jaccard) [Figure 8D and E]. Using the default parameters settings, most tools were able to cluster the samples as expected (cluster purity > 0.8), with the exception of Sourmash (cluster purity = 0), as the default sketch size parameters were too small to allow for a correct approximation of the sample’s distances. Additionally, CAFE was not able to recapitulate the expected data structure using the Cosine or D2Star distance metric and a k-mer size of 5pb (cluster purity < 0.5 for all conditions tested) [Supplementary Figure 8]. The cluster purity metrics obtained for all tools are available in Supplementary Table 1.

**Figure 9:**
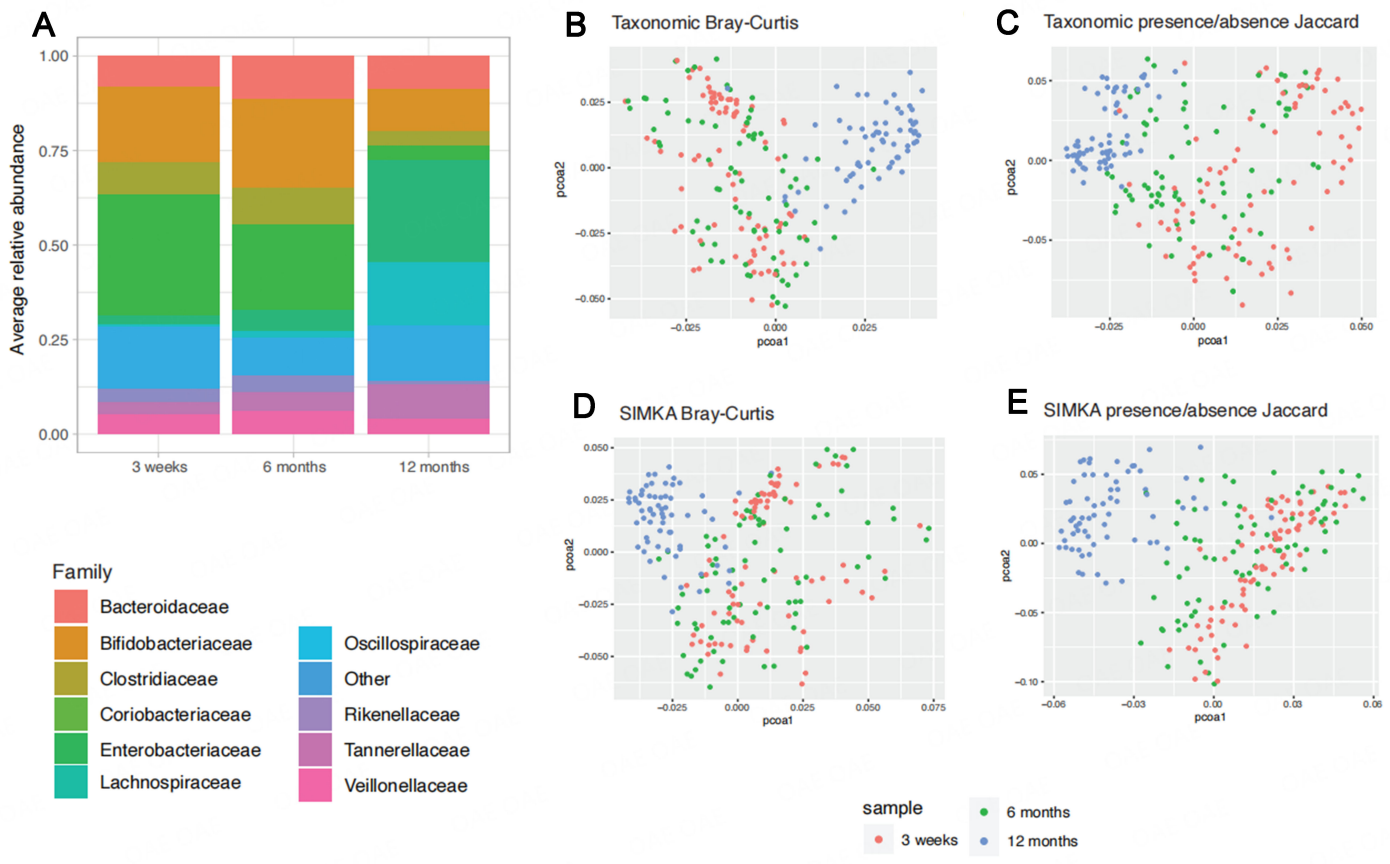
Comparison of taxonomic and k-mer-based approaches on a large dataset of infant fecal metagenomes. (A) Average composition of the samples grouped by sample origin at the Family level, taxonomic families with a prevalence below 10% and a relative abundance below 5% were grouped as “Other”; (B) PcoA of the samples on the taxonomic profiles at the species level using a Bray-Curtis distance; (C) PcoA of the samples on the taxonomic profiles at the species level using a presence/absence Jaccard distance; (D) PcoA of the samples on the k-mer spectra using a Bray-Curtis distance (E) PcoA of the samples on k-mer spectra profiles using a presence/absence Jaccard distance.

Finally, we compared the tools’ distances obtained on a large dataset of 224 samples from infant fecal microbiota sampled at 3 weeks, 6 months, and 12 months of age. These fecal samples were selected as they enable the assessment of the tools on a gradient of microbiota change [Figure 9A]. The tool’s ability to recapitulate the gradient data structure was assessed using PCoA visualizations and PERMANOVA testing. Using taxonomic annotation, both Bray-Curtis and presence/absence Jaccard distances were able to distinguish the sample time points (PERMANOVA *P* < 0.001, permutations = 999) [Figure 9B and C]. k-mer-based distances were computed for these samples as previously, with the exception of Commet, which could not scale to this larger dataset size. As observed in the small benchmark, the data structure observed using k-mer-based Bray-Curtis or presence/absence Jaccard distances recapitulates well the taxonomic data structure [Figure 9D and E]. Using the default parameters settings, most tools were able to separate the samples by age (PERMANOVA *P* < 0.001, permutations = 999), except for SimkaMin using the presence/absence Jaccard distance, whose default sketch size was not appropriate for this dataset. Additionally, CAFE was not able to recapitulate the expected data structure for all tested distances using a k-mer size of 5bp (PERMANOVA *P* > 0.001, permutations = 999) [Supplementary Figure 9].

## DISCUSSION

A central task in the analysis of metagenomic samples is the ability to compare microbial communities from different samples. Comparative metagenomics analysis typically includes measuring a distance, often an ecological beta-diversity distance between pairs of metagenomes, and the resulting distance matrix can be used for various tasks, such as visualization, clustering, or retrieval. *De novo* comparative metagenomic approaches aim to allow metagenomic comparisons in conditions where Reference-based methods are impossible given high novelty or bias due to the underrepresentation of taxa in a reference database. These computational approaches compare metagenomic samples solely on their k-mer composition, thus bypassing the need for taxonomic profiling. These types of approaches, which take into account both known and unknown taxa in the microbiota, are particularly relevant when analyzing understudied ecosystems where microbial unknowns are prevalent^[27]^.

Very few previous studies have compared taxonomic Beta-diversity metrics to k-mer-based distances. Notably, Dubinkina *et al*. explored the relationship between k-mer-based and taxonomy-based beta-diversity measurements, using simulated metagenomic datasets composed of ten human gut bacteria^[5]^. Using these simple simulated datasets, the authors reported a high correlation between taxonomy-based and k-mer-based Bray-Curtis distances (rho = 0.88 with *k* = 10bp) and observed that the correlation increases with longer k-mer sizes. Importantly, due to computational constraints, the authors only explored these correlations for a small k-mer size (maximum 12bp). These observations were later confirmed using a k-mer size above 21bp by Benoit *et al*., who demonstrated a strong correlation (rho = 0.885 with *k* = 21pb) between k-mer-based and taxonomic-based Bray-Curtis metrics on real metagenome datasets from the Human Microbiome Project (HMP)^[10]^. In this study, we built on these prior works and used simulated metagenomes to extensively compare the correlation between taxonomy-based and k-mer-based beta-diversity distances. We focused our analysis on two commonly used ecological metrics: the quantitative Bray-Curtis index and the presence/absence Jaccard index. As previously observed by Dubinkina *et al*., the correlation between taxonomic and k-mer-based beta-diversity distances improved when the k-mer length increased and reached a plateau for k-mer lengths above 20bp^[5]^.

Using simulated metagenomic datasets of increasing sequencing depth, we showed that the correlation between taxonomic and k-mer-based distances was strongly impacted by the number of reads in the metagenomes. The correlation increased with the sequencing depth, and k-mer-based distances measured between shallow metagenomes were close to 1 (completely dissimilar). This result suggests that k-mer-based distances at shallow sequencing depth tend to overestimate the dissimilarity between metagenomes. This is further confirmed when comparing the results obtained for communities of increased richness. While a strong correlation between the expected taxonomic and k-mer-based Bray-Curtis distance was measured at a sequencing depth of 5M reads for simple communities of 25 organisms, the correlation dropped in the same conditions for more complex communities composed of 500 organisms.

While sequencing depth and community richness had a notable impact on the correlation between expected and k-mer-based distances, no major impact was found for the sequencing technology and low abundance sequence contaminations. These experiments demonstrate a global resilience of k-mer-based distances towards low k-mer noise. This is in accordance with prior experiments, showing that low-rate SNP mutation had a minor impact on the k-mer-based distances^[5]^. Additionally, we assessed the effect of community phylogenetic richness on the k-mer-based distances. This experiment showed little impact of the phylogenetic richness when considering long k-mer size above 20bp.

Even if *de novo* k-mer-based methods are globally scalable, applying these methods to very large metagenomic projects containing thousands of metagenomes is still a computational challenge. In order to reduce the computational time of k-mer-based comparisons, several tools choose to approximate pairwise distances by subsampling the k-mer space, instead of considering the billions of k-mers typically present in metagenomic projects. Here we show that these sketched approaches allow for a robust estimation of the k-mer-based distance at a sketch size of 1 million k-mers or above. Importantly, the estimation is more precise for quantitative-based distances such as the Bray-Curtis metric than for presence/absence distances.

Finally, we assessed the impact of k-mer filtering on the k-mer-based distance computed between samples in a simulated metagenomic dataset. Filtering of low-abundance k-mers was proposed as a solution to palliate sequencing errors, while the filtration of high-abundance k-mers aims to remove potential sequence contaminants. Additionally, filtering out the rare k-mers reduces the computational requirements of the comparison by reducing the number of unique k-mers to be taken into account^[10]^. In the conditions chosen for the simulated experiment (simple mock communities of 25 organisms each, sequenced at 5 million reads), applying a low abundance k-mer filter consistently degraded the correlation between expected and k-mer-based distances, even for a k-mer minimum abundance filter of 2. Importantly, while our simulated metagenomes allow for realistic modeling of sequencing errors, we acknowledge that additional sequencing errors not simulated in our experiments could be present in real metagenomic datasets. As expected, the potential impact of k-mer filtering is particularly important to consider when using distances such as the presence/absence Jaccard distance.

To our knowledge, there are 12 tools currently published for k-mer-based *de novo* comparative metagenomic tasks. Older tools, such as Commet, TriageTool, and Compareads, used k-mers to compare metagenomes in terms of read content. However, these approaches are unable to scale to modern metagenomic dataset sizes. More recent approaches compare datasets on their k-mer content directly. We benchmarked all *de-novo* comparative metagenomic tools that could be installed and run on a dataset of 30 metagenomes. Most tools were able to compute pairwise distances in less than 5 h. Strikingly, Simka allowed for a comparison of the samples on their complete k-mer spectra in less than 3 h, a run time comparable to other tools such as Mash or Sourmash that use a sketching approach. The fastest tool in this benchmark was SimkaMin, which was able to perform the comparison in less than 30 minutes. Finally, we compared the output of all tools on two real metagenomic datasets, and assessed if the tools were able to recapitulate data structures observed taxonomically. Importantly, most of the tested k-mer-based *de novo* tools were able to successfully recapitulate this data structure using the standard parameters, with the exception of CAFE, whose recommended small k-mer size (5-13bp) seems not to be appropriate for a fine-scale exploration of metagenomic differences.

### Recommendations for *de novo* comparative metagenomic users

From the experiments and benchmarks performed in this study, we highlight key points for users interested in applying *de novo* comparative methods to their metagenomic datasets. In terms of usability, ease of installation, and computational requirements, we believe that Simka allows for a fast and accurate k-mer-based comparison of metagenomic datasets, and SimkaMin provides an alternative for the fast estimation of Bray-Curtis and presence/absence Jaccard distances for very large-scale datasets or for users with limited computational resources.

In accordance with previously published observations, we recommend using a k-mer length of 20bp or above to measure k-mer-based Bray-Curtis distances between metagenomes, in order to obtain results that are well correlated with taxonomic-based distances. However, we highlight here that presence/absence k-mer-based metrics such as the presence/absence Jaccard do not correlate well with the equivalent taxonomic-based distances. Importantly, our experiments also show that sequencing depth can have a drastic effect on the k-mer-based distances, and users should look out for inflation of k-mer distances close to or equal to 1. Finally, users should limit their use of minimum abundance k-mer filters to cases where they strongly suspect a large number of erroneous k-mers, or in case of computational limitations. However, in this situation, the users should refrain from using presence/absence distances, as they are most affected by the filtration of k-mers.

### Limitations and future directions

This study aimed to provide an overview of the current k-mer-based *de novo* comparative approaches, evaluating their strengths and current limitations. Here, we highlight additional limitations and future research directions that are particularly interesting, although outside the scope of this current study. In particular, our study focused on k-mer-based tools and showed their applicability to short-read sequencing metagenomes. Importantly, k-mers fail to distinguish between similar sequences arising from high sequencing error rate. Error tolerance is particularly important for long reads technologies (Oxford Nanopore Technologies or Pacific Biosciences of California sequencing platforms). To allow a deterministic level of tolerance for base mismatches, several authors have proposed to replace k-mers with spaced seeds^[28,29]^. Additionally, we focused here on tools performing comparative metagenomic tasks, but further studies should also include additional k-mer-based tools, such as KmerGo, which capture group-specific k-mers between groups of metagenomic sequencing datasets^[30]^.

## DECLARATIONS

### Availability of data and materials

The compositions of each sample of the simulated datasets are available in Supplementary File 1. The taxonomic classification and NCBI ID of the genomes used are available in Supplementary File 2. The pipeline used to generate the mock metagenomes is available on GitHub at: https://github.com/hurwitzlab/Simulated_metagenomes_generator. The list of publicly available metagenomic samples included in the benchmark is available in Supplementary File 3. The code used to run tools is available on GitHub at: https://github.com/mattmiller899/de_novo_metagenomic_tools. The parameters used to run each tool for the benchmark experiment is listed in Supplementary Table 2.

### Copyright

© The Author(s) 2023.
