# Supplementary material for: Comparison of k-mer-based de novo comparative metagenomic tools and approaches
Source: Microbiome Res Rep. 2023 Jul 20;2(4):27. doi: 10.20517/mrr.2023.26 (PMC10696585; doi:10.20517/mrr.2023.26)
Supplement: Supplementary file 1 [file mrr-2-4-27-SupplementaryMaterials.pdf]

Supplementary Figures

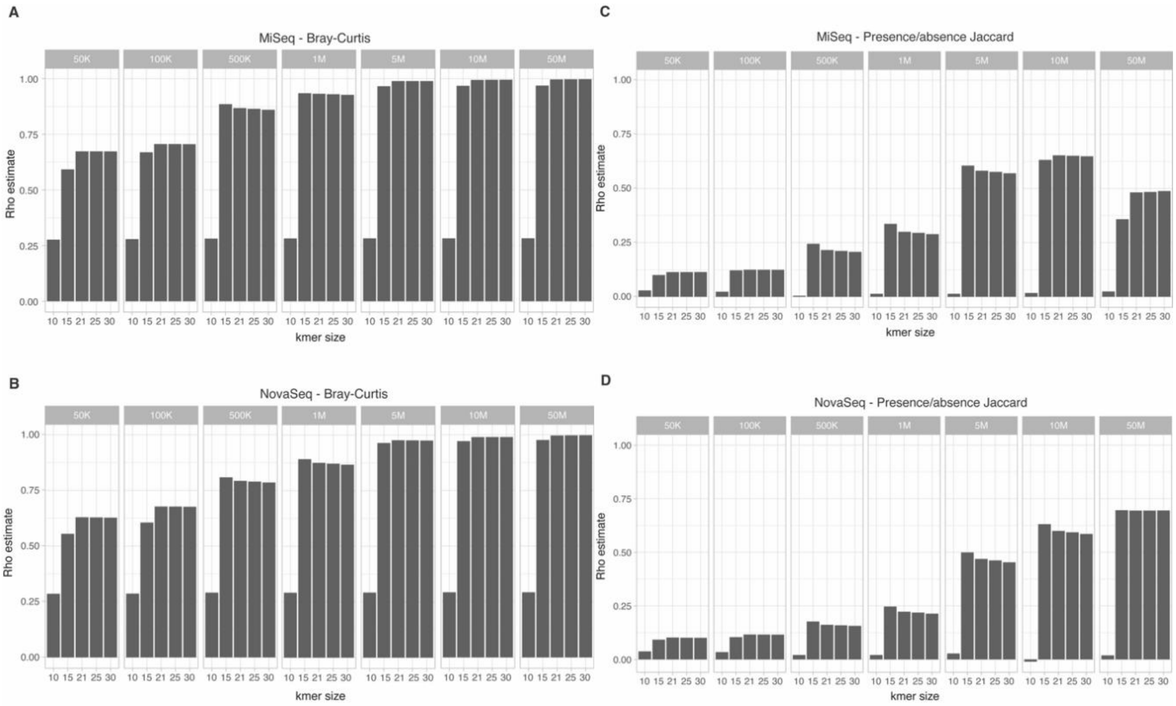

Supplementary Figure 1. Impact of Sequencing technology on the k-mer based beta-diversity distances. (A) Spearman correlations between the expected taxonomic and k-mer based Bray-Curtis distance using the MiSeq sequencing error model; (B) Spearman correlations between the expected taxonomic and k-mer based Bray-Curtis distance using the NovaSeq sequencing error model; (C) Spearman correlations between the expected taxonomic and k-mer based presence/absence Jaccard distance using the MiSeq sequencing error model; (D) Spearman correlations between the expected taxonomic and k-mer based presence/absence Jaccard distance using the NovaSeq sequencing error model.

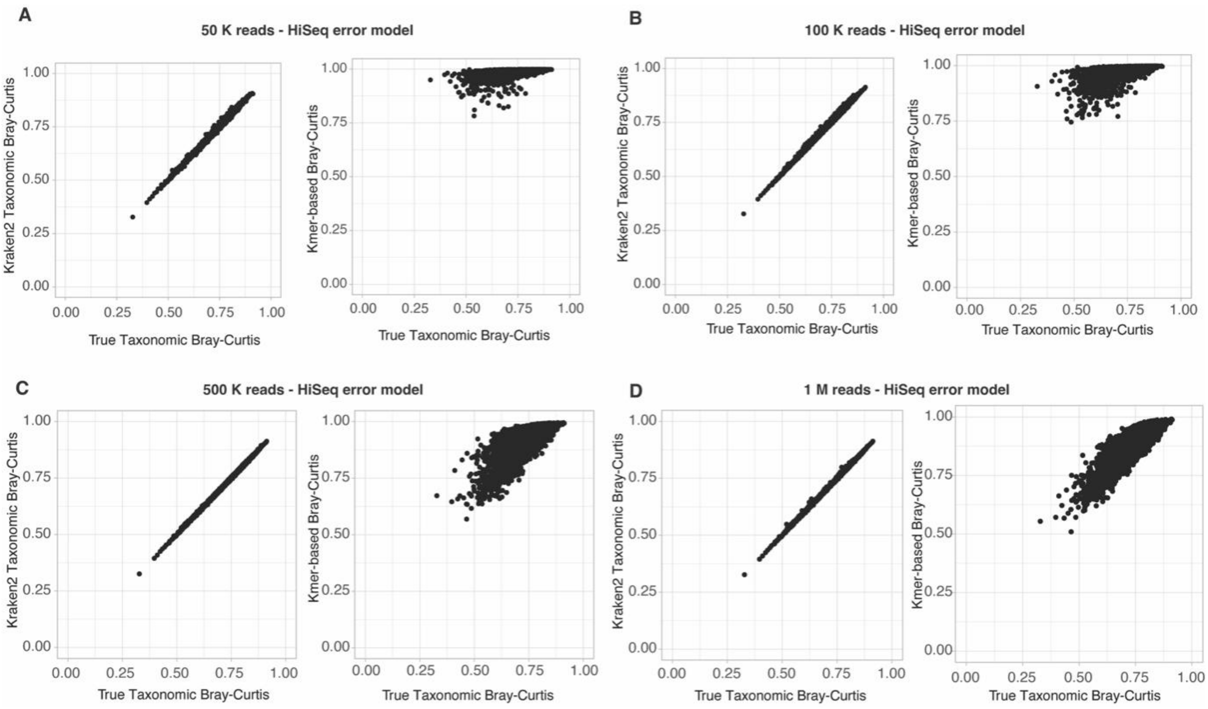

Supplementary Figure 2. Impact of Sequencing depth on k-mer based beta-diversity distances. Expected taxonomic against the read-based taxonomic distances or against the k-mer based (k = 20bp) Bray-Curtis distances at a sequencing depth of (A) 50K paired reads; (B) 100K paired reads; (C) 500K paired reads and (D) 1 Million paired reads using the HiSeq sequencing error model.

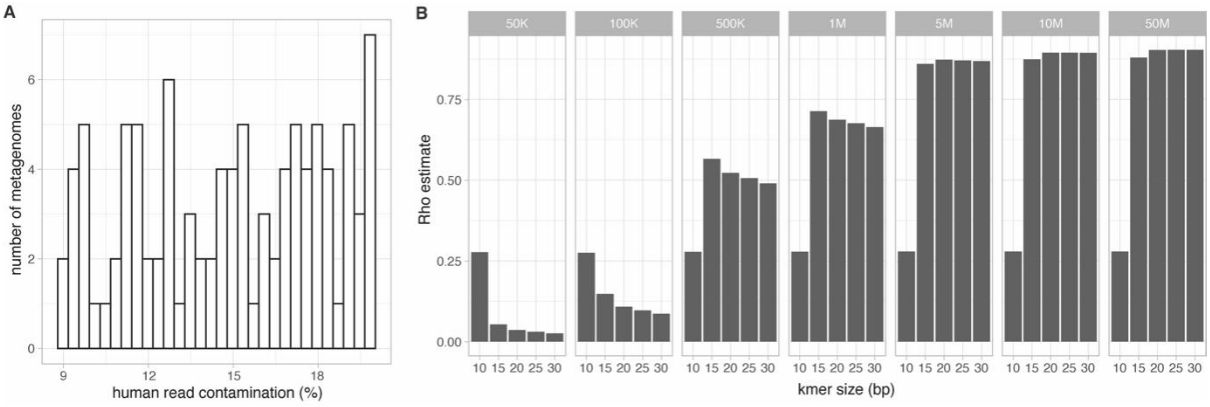

Supplementary Figure 3. Impact of high abundance human sequence contaminations on the correlation between expected taxonomic and k-mer based beta-diversity metric. (A) Distribution of the percentage of human reads content in the high contamination simulated dataset; (B) Spearman correlations between the expected taxonomic and k-mer based Bray-Curtis distance for the high contamination human contaminated dataset.

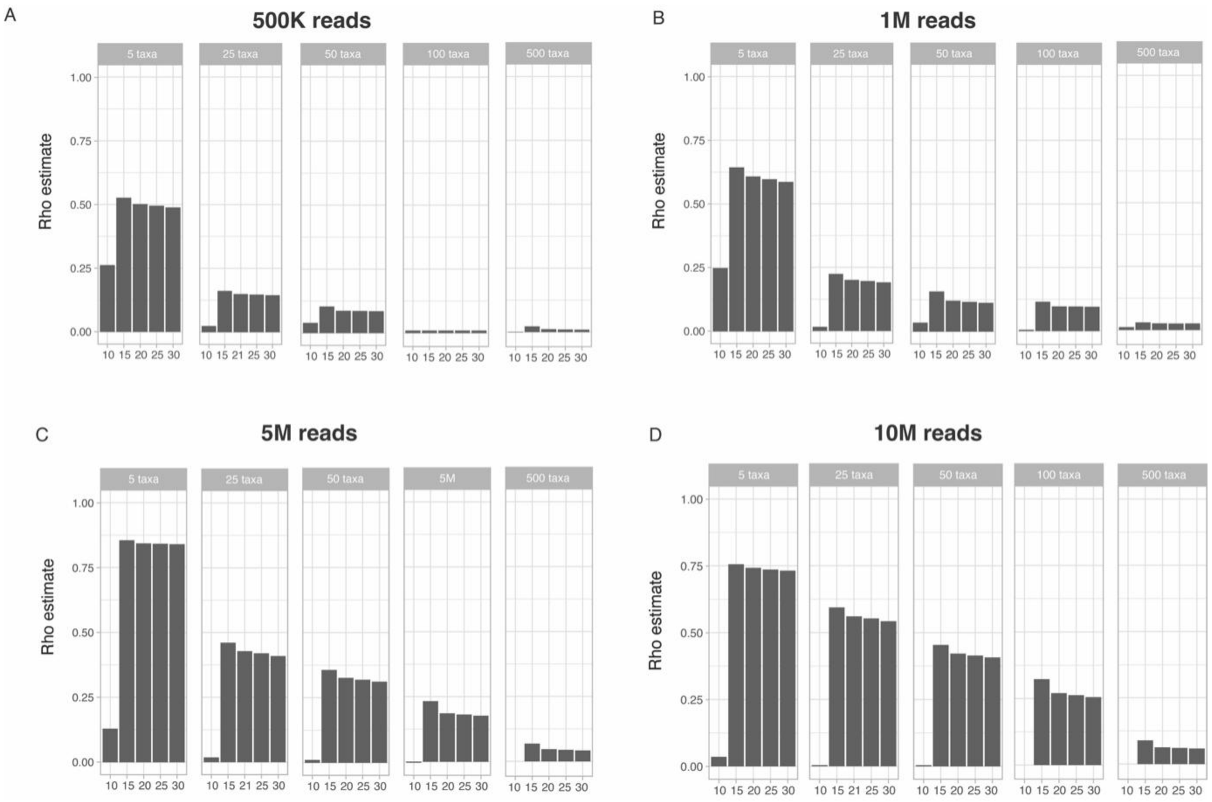

Supplementary Figure 4. Impact of increasing community species richness on the correlation between expected taxonomic and k-mer based presence/absence Jaccard metric. Spearman correlations between the expected taxonomic and k-mer based presence/absence Jaccard distance for simulated communities containing an increasing number of taxa, for a simulated sequencing depth of (A) 500K paired-reads; (B) 1 Million paired-reads; (C) 5 Million paired-reads or (D) 10 Million paired-reads.

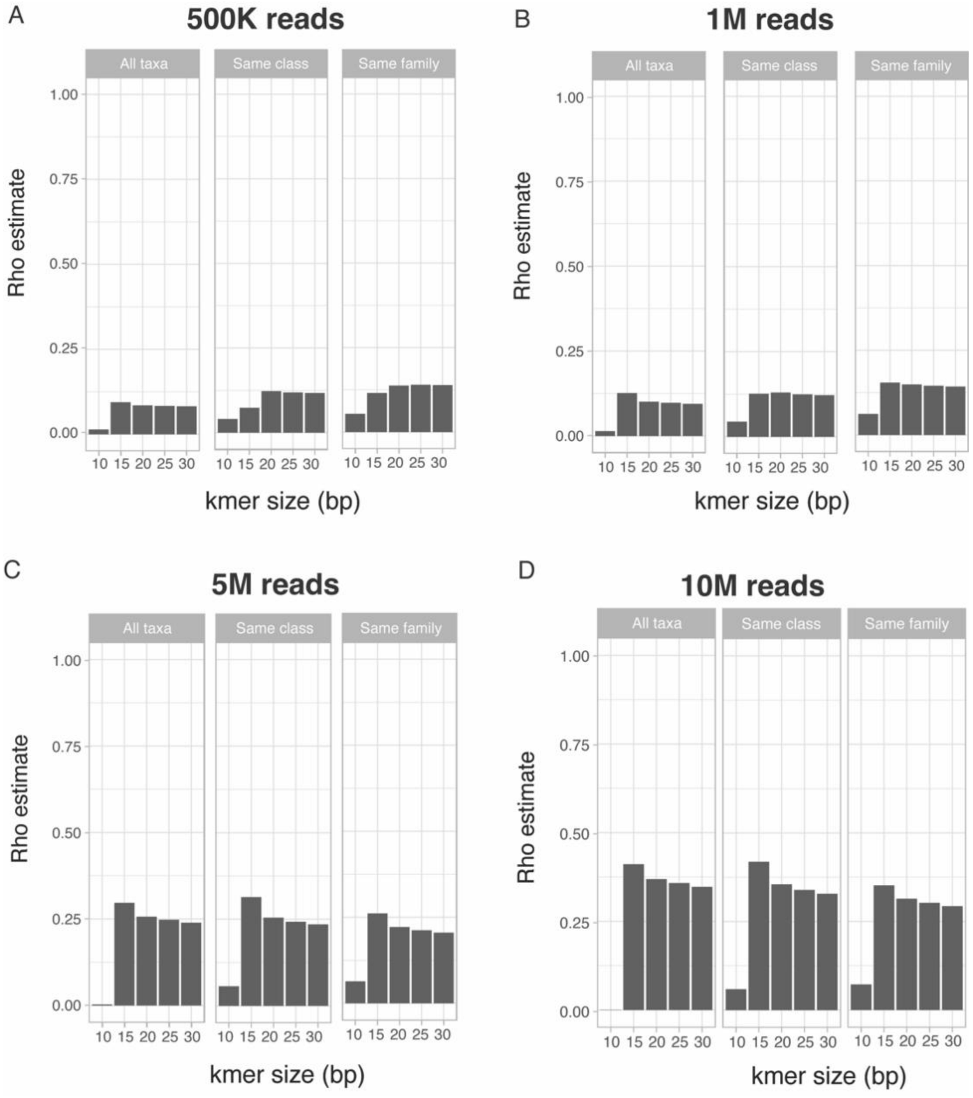

Supplementary Figure 5. Impact of decreasing taxonomic diversity on the correlation between expected taxonomic and k-mer based presence/absence Jaccard metric. Spearman correlations between the expected taxonomic and k-mer based presence/absence Jaccard distance for simulated communities containing 50 taxa from all possible taxonomic classes ("All taxa"), from the Actinomycetes class ("Same class") or from the Mycobacterium family ("Same family"). Simulated metagenomes were generated to simulate a sequencing depth of (A) 500K paired-reads; (B) 1 Million paired-reads; (C) 5 Million paired-reads or (D) 10 Million paired-reads.

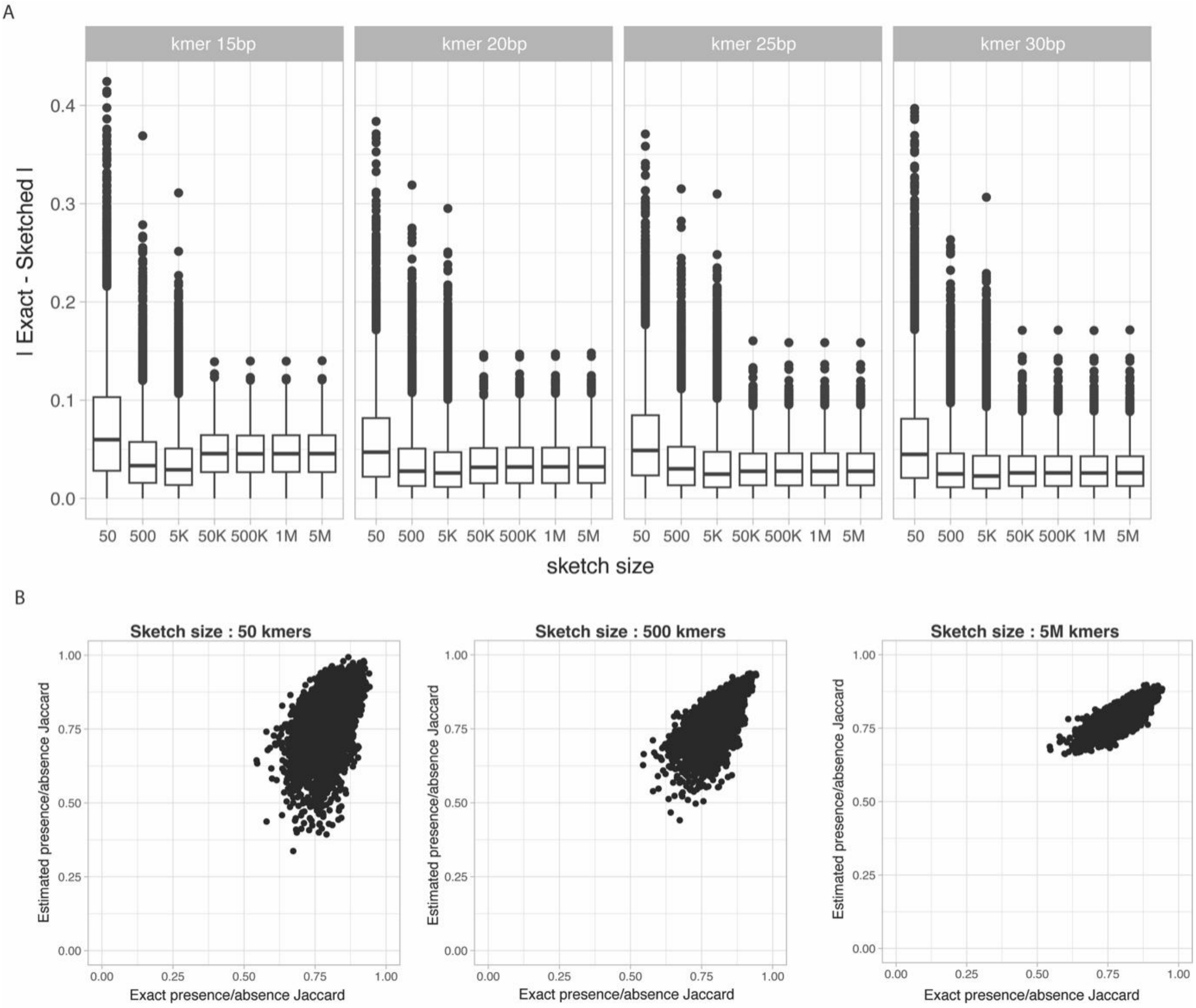

Supplementary Figure 6. Impact of sketching k-mer on the estimation of k-mer based presence/absence Jaccard distances. (A) Absolute differences between the exact k-mer based and sketched presence/absence Jaccard distances for an increasing Sketch size; (B) Exact k-mer-based against the sketched presence/absence Jaccard distances (k = 30bp) obtained for a simulated dataset of 100 metagenomes simulated at a sequencing depth of 5 million paired-reads using the HiSeq sequencing error model.

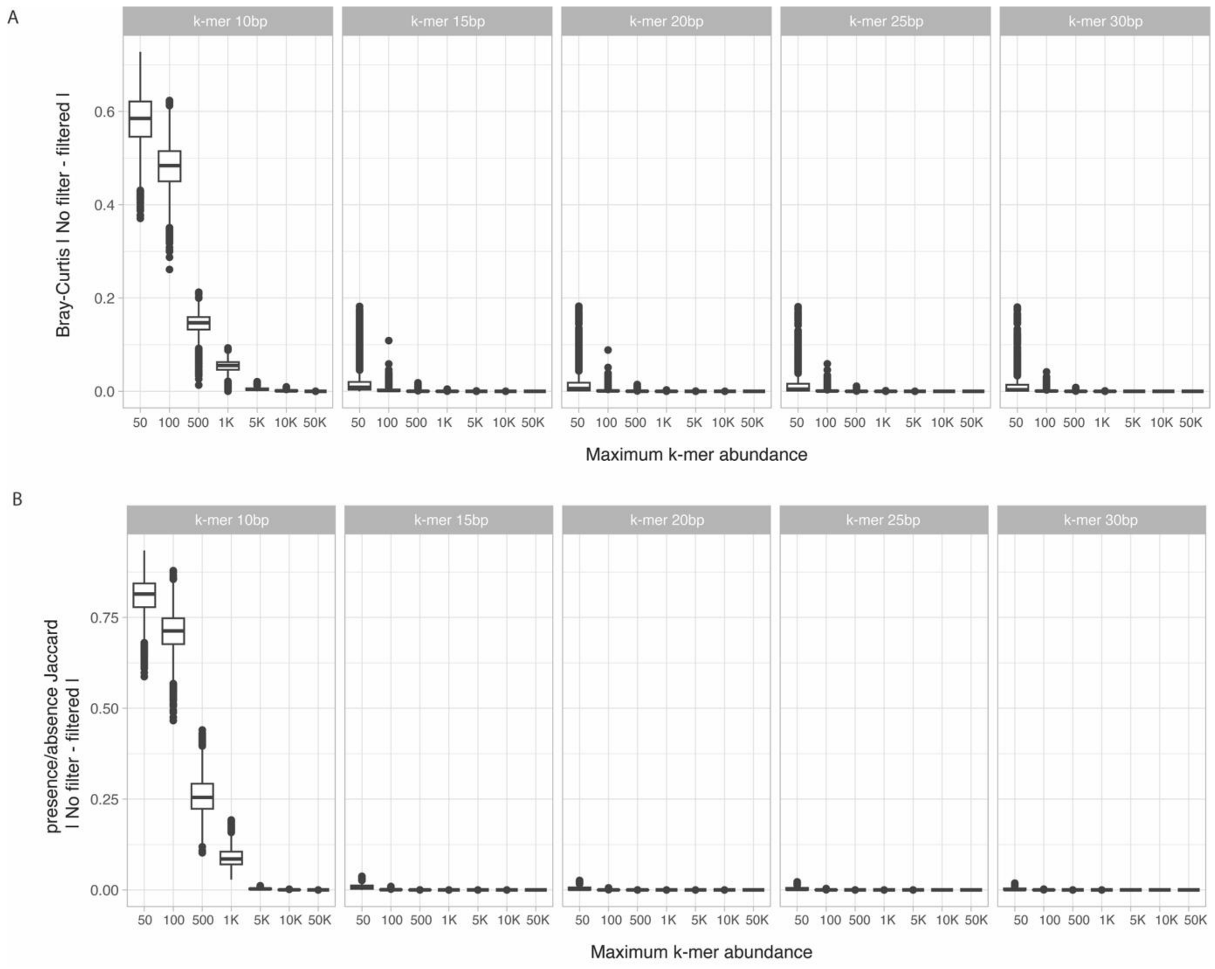

Supplementary Figure 7. Impact of high abundance k-mer filter on the estimation of k-mer based Bray-Curtis distances. (A) Absolute differences between the exact k-mer Bray-Curtis distances and distances after high abundance k-mer filter; (B) Absolute differences between the exact k-mer presence/absence Jaccard distances and distances after high abundance k-mer filter.

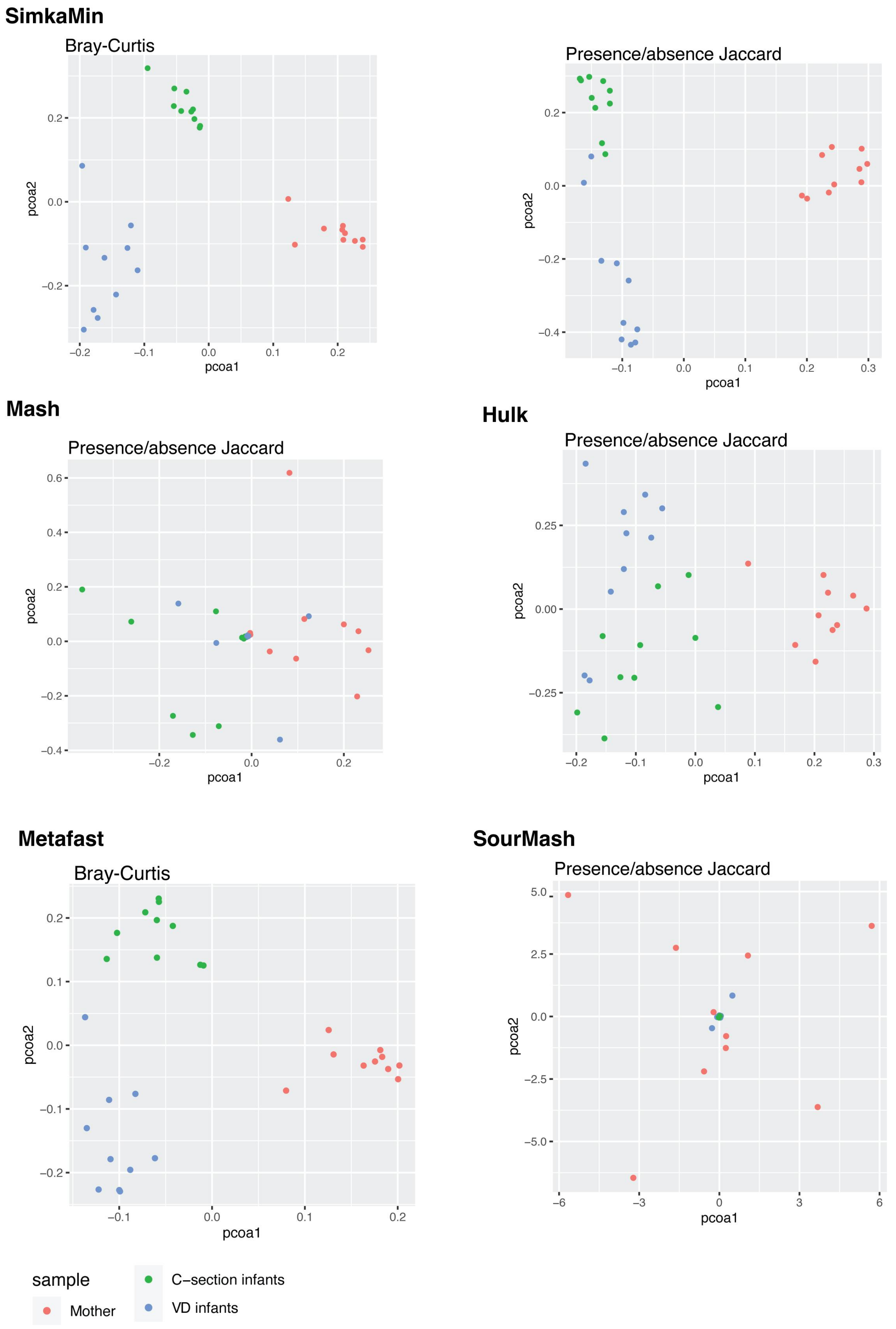
SimkaMin
Bray-Curtis
Presence/absence Jaccard
Mash
Presence/absence Jaccard
Hulk
Presence/absence Jaccard
Metafast
Bray-Curtis
SourMash
Presence/absence Jaccard
pcoa1
pcoa2
sample
Mother
C−section infants
VD infants

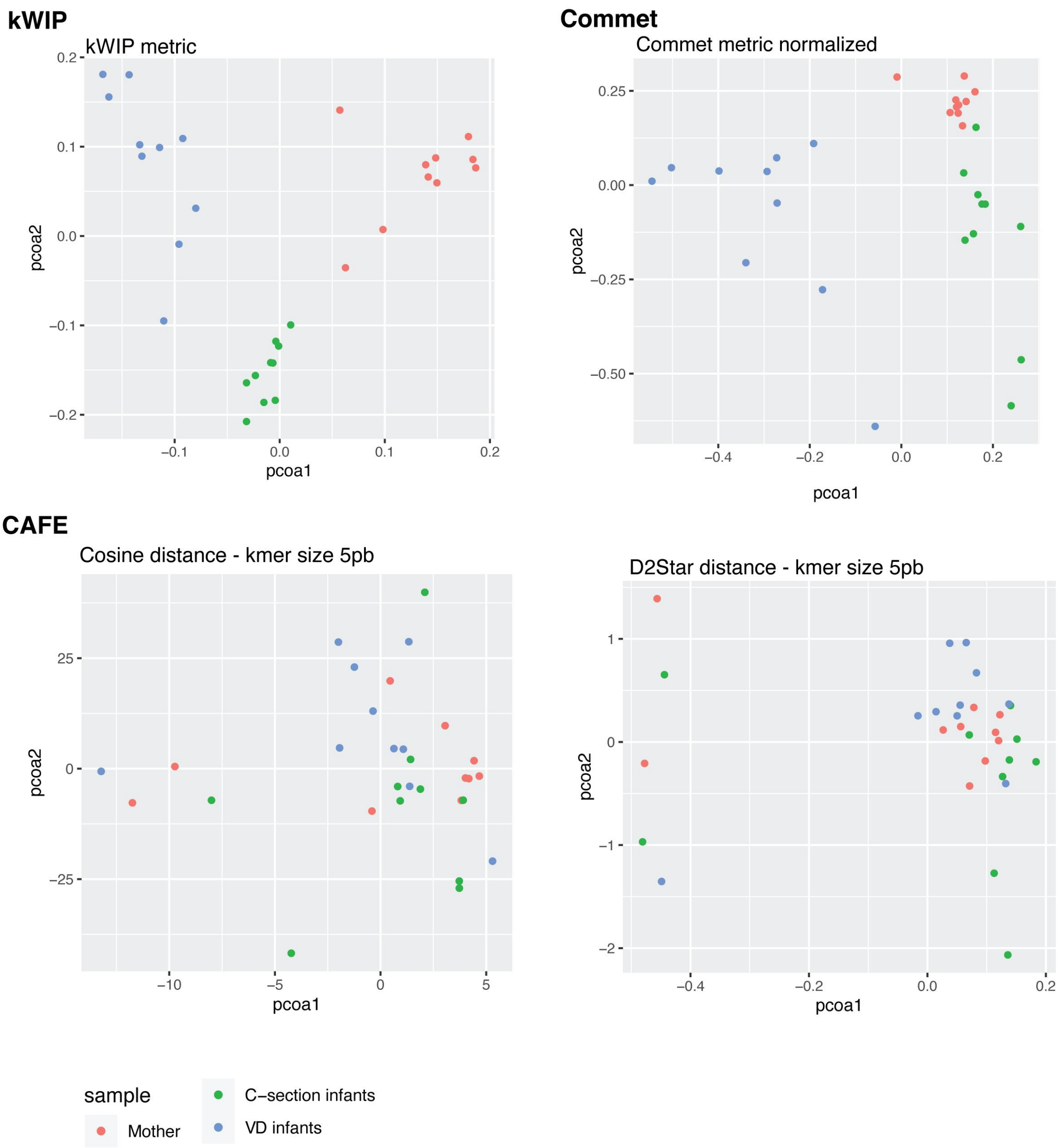

Supplementary Figure 8. Comparison of taxonomic and k-mer based approaches on a small dataset of infant and maternal fecal metagenomes. PcoA of the samples on k-mer spectra profiles were computed using each tool's provided distance metric for a k-mer size of 31bp, except CAFE that was run using a k-mer size of 5bp, and using each tool's default parameters.

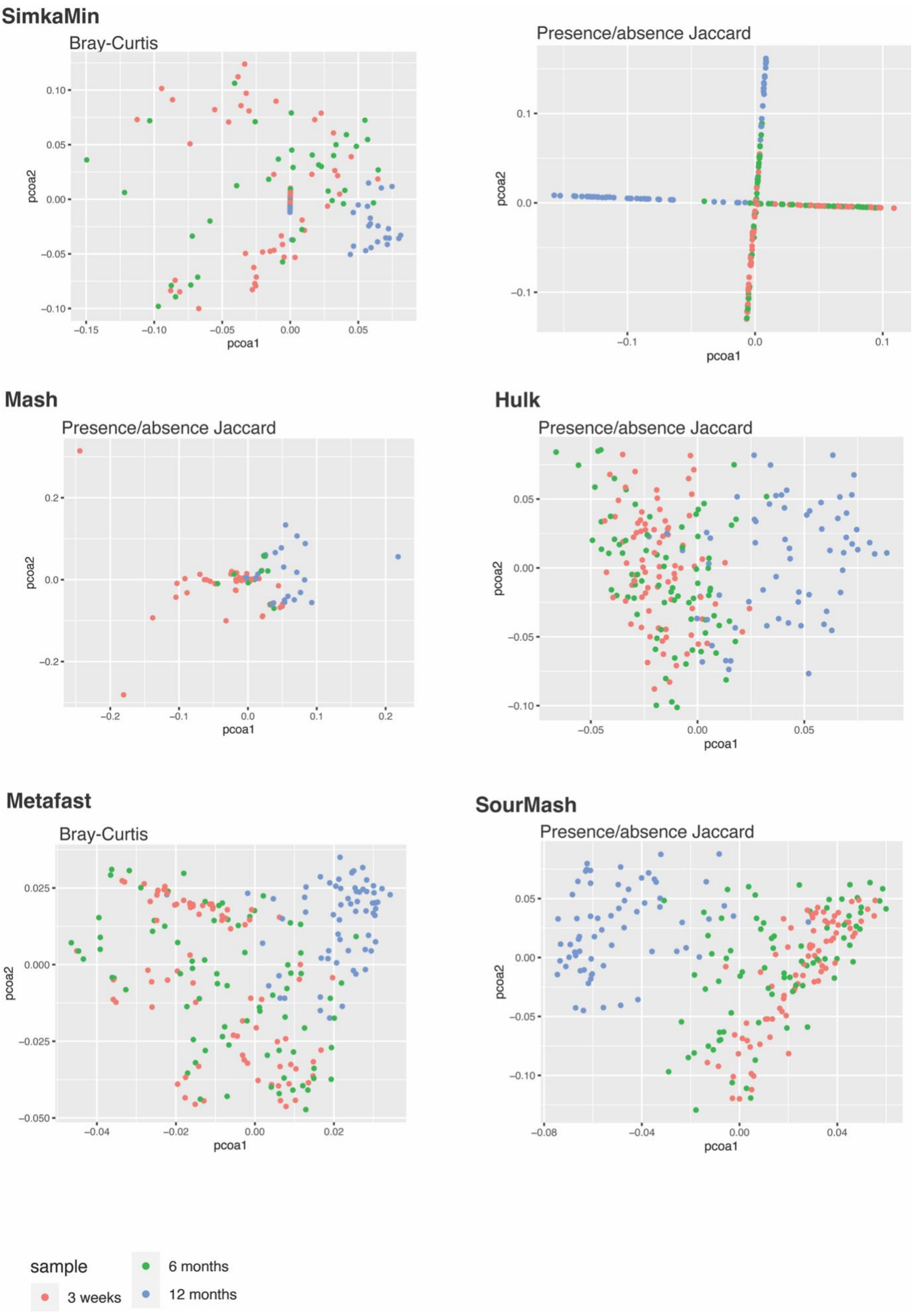
SimkaMin
Bray-Curtis
Presence/absence Jaccard
Mash
Presence/absence Jaccard
Hulk
Presence/absence Jaccard
Metafast
Bray-Curtis
SourMash
Presence/absence Jaccard
pcoa1
pcoa2
sample
3 weeks
6 months
12 months

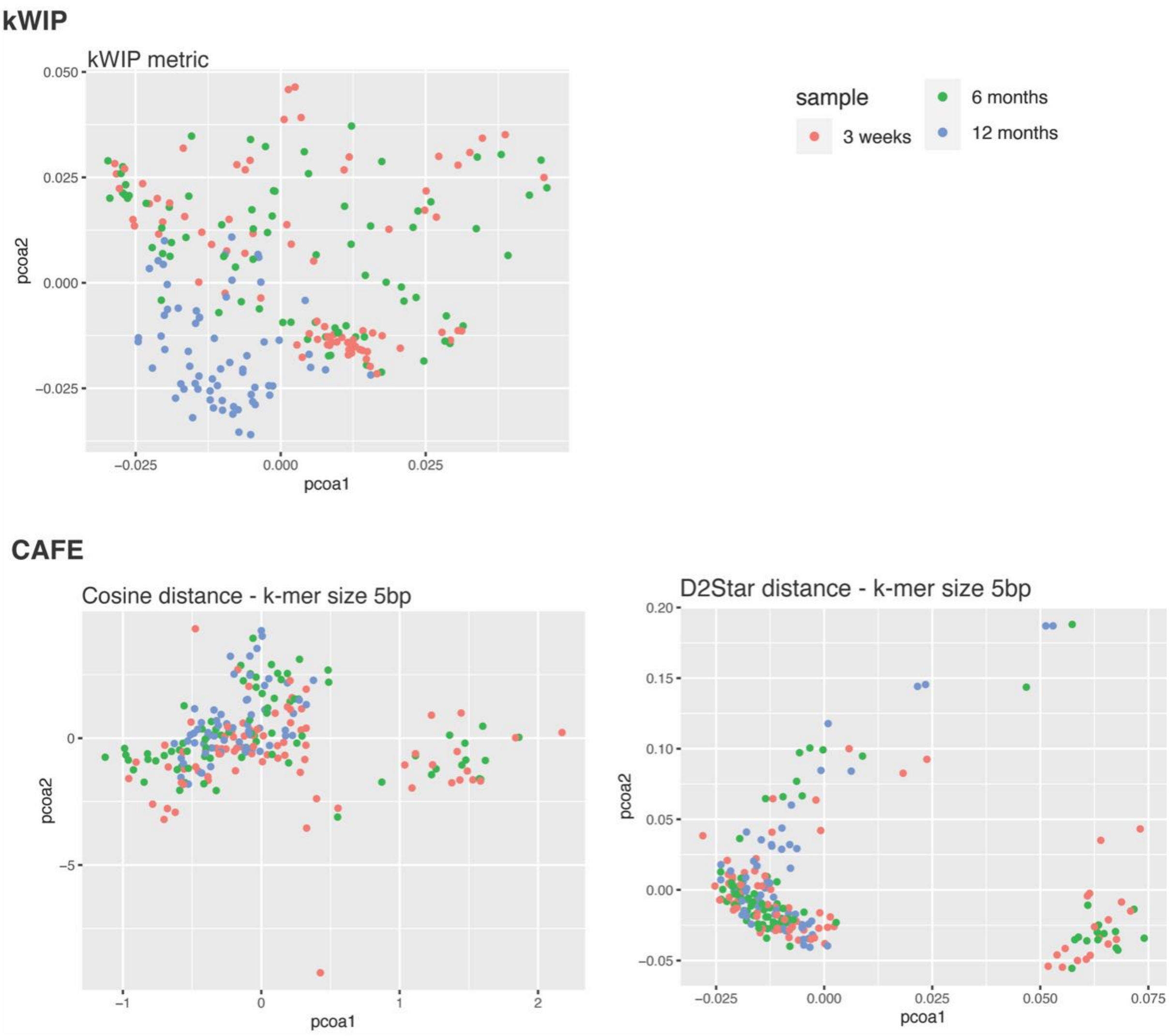

Supplementary Figure 9. Comparison of taxonomic and k-mer based approaches on a large dataset of infant fecal metagenomes. PcoA of the samples on k-mer spectra profiles were computed using each tool's provided distance metric for a k-mer size of 31bp and using each tool's default parameters.

Supplementary table 1. Cluster purity obtained on a small dataset of fecal metagenomes

Software	Cluster purity
CAFE (31bp)	Cosine = 0.46, D2S = 0.4
Commet	0.93
Hulk	0.8
kWIP	0.97
mash	0.9
metafast	0.9
Simka	BC = 0.97, JC = 0.93
SimkaMin	BC = 0.97, JC = 0.9
sourmash	0 (One unique cluster)

Each tool was run using the provided distance metric for a k-mer size of 31bp and using each tool's default parameters. Hierarchical clustering was performed using Ward's method, and the cluster purity was calculated on the obtained cluster. BC: Bray-Curtis; JC: presence/absence Jaccard; D2S: D^2Star distance.

Supplementary Table 2. Parameters used to run each tool during the benchmark comparison

Software	commands and parameters
CAFE	"./cafe -M 5 -O $OUT_DIR -I $JELLYFISH -K 5 -D D2star,Cosine"
Commet	"python commet/Commet.py $FILE_LIST -b commet/bin -o $OUT_DIR -k 31 -m 10000"
Hulk	"hulk sketch -p 4 -k 31 -o $OUT_DIR/$SKETCH_FILE -f $IN_FILE"<br>"hulk smash -p 4 -k 31 -o $OUT_FILE -d $OUT_DIR"
kWIP	"python khmer/load-into-counting.py $OUT_DIR/$COUNT_FILE $IN_FILE -k 31 -T 4 -N 1 -x 1e10"<br>"kwip -t 4 -k $KERNEL_OUT -d $DIST_OUT $OUT_DIR/*.ct"
mash	"mash sketch -l $FILE_LIST -o $SKETCH -p 4 -k 31"<br>"mash dist $SKETCH -l $FILE_LIST -p 4 -t > $OUT_FILE"
metafast	"./metafast.sh -i \`cat $FILE_LIST\` -k 31 -m 160G -p 4 -w $OUT_DIR"
Simka	"simka -kmer-size 31 -in $FILE_LIST -out $OUT_DIR -out-tmp $TMP_DIR -nb-cores 128 -max-memory 768000 -count-file ./simka_count.sh -merge-file ./simka_merge.sh -count-cmd 'sbatch --partition=standard --account=$ACCT --mail-user=$EMAIL --mail-type=FAIL' -merge-cmd 'sbatch --partition=standard --account=$ACCT --mail-user=$EMAIL --mail-type=FAIL' -max-count 32 -max-merge 32"
SimkaMin	"simkaMin.py -kmer-size 31 -in $FILE_LIST -out $OUT_DIR -nb-cores 4 -max-memory 24000"
sourmash	"sourmash sketch dna \`cat $FILE_LIST\` –outdir $SKETCH_DIR -p k=31"<br>"sourmash compare $SKETCH_DIR/*.sig -p 4 -o $OUT_DIR/out.dist –csv $OUT_DIR/out.csv"

$File_LIST: path to list of input files; $OUT_DIR: output directory path; $SKETCH_DIR: path to intermediary directory to store sketching files; $ACCT: SLURM user account; $EMAIL; email of the SLURM user.
